# Iron-Loaded Carbon Spherogels as Sustainable Electrode Materials for High-Performance Lithium-Ion Batteries

**DOI:** 10.1021/acs.chemmater.5c02442

**Published:** 2026-01-29

**Authors:** Saeed Borhani, Le Thi Thao, Gregor A. Zickler, Antje Quade, Michael S. Elsaesser, Volker Presser, Stefanie Arnold

**Affiliations:** † Chemistry and Physics of Materials, 27257 University of Salzburg, Salzburg 5020, Austria; ‡ 28391 INM - Leibniz Institute for New Materials, Campus D2 2, Saarbrücken 66123, Germany; § Department of Materials Science & Engineering, 9379 Saarland University, Campus D2 2, Saarbrücken 66123, Germany; ∥ 28372 Leibniz Institute for Plasma Science and Technology, Felix-Hausdorff-Straße 2, Greifswald 17489, Germany; ⊥ Saarene - Saarland Center for Energy Materials and Sustainability, Campus C4 2, Saarbrücken 66123, Germany

## Abstract

The increasing demand for sustainable energy storage drives the development of advanced lithium-ion battery (LIB) materials that combine high performance, cost efficiency, and environmental sustainability. Carbon spherogels, characterized by high surface area, interconnected porosity, and high conductivity, are promising electrode candidates; however, they suffer from low specific capacities when used alone. This study presents iron-loaded carbon spherogels as next-generation LIB electrodes, leveraging iron’s high theoretical capacity, abundance, and eco-friendliness. A scalable and tailorable synthesis method enabled the integration of tunable iron contents (15–40 mass %) into the carbon framework, forming robust porous networks with uniformly distributed iron nanoparticles. Electrochemical characterization revealed high specific capacities (up to 1190 mAh g^–1^) and high cycling stability (>99% Coulombic efficiency over 300 cycles). Post-mortem analysis highlighted the synergistic interaction between iron redox activity and carbon matrix stability. The medium (27 mass %) iron-loaded carbon spherogel sample achieved the best balance between capacity and durability. These findings position iron-loaded carbon spherogels as sustainable, high-performance LIB electrodes, offering a cobalt-free and nickel-free alternative that addresses key challenges of conversion-type materials, such as volume expansion and capacity fading.

## Graphical abstract

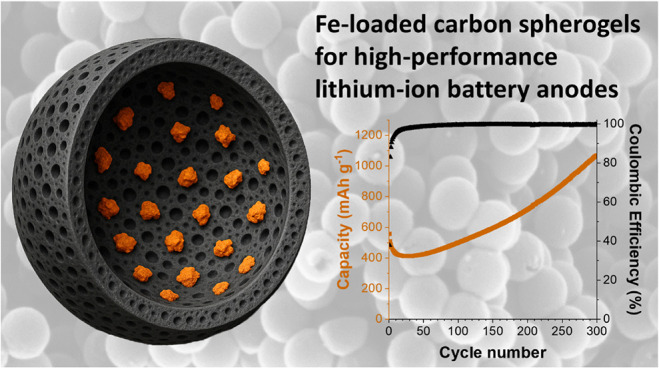

## 1 Introduction

The increasing demand for sustainable energy storage systems has placed lithium-ion batteries (LIBs) at the forefront of energy research.^1^ With their widespread application in portable electronics, electric vehicles, and renewable energy integration, LIBs require continual innovation to improve their performance, safety, and environmental footprint.^2^ However, the reliance on critical and costly materials, such as cobalt and nickel in conventional cathode chemistries, poses significant challenges, including resource scarcity, ethical mining concerns, and high production costs.^3^ This has spurred the exploration of alternative, sustainable electrode materials that can deliver high performance while addressing environmental and economic concerns.

Carbon-based materials have emerged as promising candidates for LIB electrodes due to their abundance, chemical stability, and tunable structural properties.^4^ Carbon spheres, in particular, synthesized via different strategies such as template assistance,^5^ sol-gel,^6^ and hydrothermal carbonization,^7^ have gained significant attention as promising materials for various energy storage applications due to their high surface area, interconnected porous structure, mechanical robustness, and high electrical conductivity.^8–10^ Carbon spherogels, as tailored porous carbon aerogels, were introduced in 2019,^9^ employing templating in the sol-gel process of resorcinol-formaldehyde. They benefit from a free-standing monolithic structure formed by a network of exclusively uniform-sized hollow carbon spheres that are adjustable in their inner hollow spheres and carbon shell thicknesses. Carbon spherogels are relevant for electrochemical energy storage applications due to their high surface area, ion-accessible hierarchical porosity, and high electrical conductivity.^8^ In general, hollow carbon sphere materials have been widely explored as electrode materials for supercapacitors, LIBs, and sodium-ion batteries, as well as for capacitive deionization.^11^ Despite their manifold advantages, pure carbon spherogels, like other pure carbon materials, face limitations in specific capacity when used as standalone LIB electrodes, necessitating hybridization with active materials to enhance their electrochemical performance. Accessible internal macropores enclosed by microporous carbon shells enable the efficient accommodation and effective integration of active materials in carbon spherogels.

Earlier efforts to hybridize carbon spherogels focused on titanium dioxide (TiO_2_), an environmentally benign and nontoxic material with good structural stability and cycling performance.^12^ However, the limited electrical conductivity, low ionic diffusion, and moderate specific capacity of TiO_2_ restricted its broader application in large-scale LIBs.^13^ To overcome these challenges, previous studies incorporated sulfur into titania-loaded carbon spherogels, creating a hybrid material with a core–shell structure.^14^ This design achieved high lithium storage capacities by leveraging sulfur’s conversion reaction and the robust framework of carbon spherogels, offering a specific capacity of 825 mAh g^–1^ after 150 cycles without requiring additional conductive additives.^14^ Such findings underscore the potential of core-shell structures for managing volume expansion, improving electron/ion transport, and stabilizing electrochemical performance in conversion-type materials.^15^ A key drawback is the shuttling effect, where soluble polysulfides cause capacity fading and reduced cycling stability.^16^ Additionally, the large volume expansion during sulfur lithiation can strain the electrode structure, leading to mechanical degradation over time.^17^ Sulfur’s low intrinsic conductivity and the difficulty of achieving uniform sulfur loading further limit the performance.^18^ The use of hazardous hydrogen sulfide during synthesis also raises safety and environmental concerns. In contrast, iron-loaded carbon spherogels could avoid these issues by offering more stable redox reactions, smaller volume changes, and better conductivity. Their synthesis is safer and more environmentally friendly, and they exhibit higher long-term cycling stability, albeit with a lower theoretical capacity compared to sulfur.^19,20^ Thus, while sulfur-based materials provide a high energy density, iron-loaded spherogels are a more robust and sustainable option for LIB electrodes.

Building on previous advancements, this study introduces iron-loaded carbon spherogels as next-generation electrode materials for sustainable, high-performance LIBs. Iron, an abundant, low-cost, and environmentally friendly transition metal, provides unique advantages for LIBs, including high theoretical capacities and redox activity.^21^ By integrating iron into the porous and conductive framework of carbon spherogels, this approach addresses common challenges of conversion-type materials, such as capacity fading and cycling instability.^19,22,23^ Combining the hierarchical porosity, lightweight nature, and high conductivity of carbon spherogels with the redox-active properties of iron, this material design aims to deliver enhanced energy and power densities, improved cycling stability, and increased safety compared with conventional electrodes. Detailed structural and electrochemical characterization further elucidates the structure property relationships of these materials, showcasing their potential to drive more sustainable and efficient energy storage solutions.

## 2 Experimental Section

### 2.1 Synthesis of the Iron-Loaded Carbon-Spherogel Material

Iron­(II) lactate hydrate (≥98.0%), styrene (≥99.0%), polyvinylpyrrolidone (PVP, average molar mass: 40,000), resorcinol (99.0% purity), formaldehyde solution (37% in water, 10% methanol as stabilizer), nitric acid (70%, reagent grade), and sodium carbonate (≥99.9%, anhydrous) were acquired from Sigma-Aldrich. Potassium persulfate (KPS, ≥99.0%) and acetone (reagent grade, ≥99.0%) were obtained from Honeywell, Fluka, and VWR, respectively. All chemicals were used without further purification.

Polystyrene (PS) nanospheres were synthesized via an emulsion polymerization reaction of styrene at 70 °C for 24 h, using PVP as the stabilizer and KPS as the initiator.^24^ The obtained white PS dispersion was washed three times by repeated centrifugation and the addition of deionized water. Before synthesis, the colloidal PS sphere solution was diluted to 9 mass % and stored at 8 °C.

In a typical synthesis procedure, three samples were prepared using 1.6, 3.2, or 4.8 g of iron lactate hydrate dissolved in 16 mL of water and added to 50 mL of the 9% PS solution under continuous stirring. After 30 min, 1.24 g of resorcinol (R) was added to the iron lactate/PS solution, followed by 10 min of additional stirring. Next, 1.83 g of formaldehyde (F) was added dropwise with gentle stirring. Afterward, 48 mg of sodium carbonate was introduced as a catalyst, followed by 5 min of stirring. The pH of the solution was adjusted from its initial value of 5.2 to 3.0 by adding 2 M nitric acid. The mixture was stirred for 60 min, and the resulting solution was filled into glass vessels and transferred to an oven at 80 °C for 5 days to facilitate gelation. After aging, the wet gels were subjected to solvent exchange by immersing them in 100 mL of acetone for three cycles over 3 days, ensuring complete solvent exchange and washing out unreacted precursor chemicals and impurities. The wet gels were then dried using supercritical CO_2_ at 11 MPa and 60 °C. Finally, the dried monolithic gels were carbonized at 800 °C under an argon atmosphere at a heating rate of 60 °C h^–1^ for 2 h, resulting in the formation of iron-encapsulated carbon spherogels.

### 2.2 Material Characterization

X-ray diffraction (XRD) for phase identification of the materials and the electrodes (pristine and post-mortem) was performed using a Bruker D8 Discover diffractometer equipped with a copper anode (CuKα, λ = 1.5406 Å, 40 kV, 40 mA). An EIGER2 two-dimensional X-ray detector was employed to record data over a range of 5°80° 2θ. The measurements were carried out in continuous mode with an angular increment of 0.019° 2θ and a counting time of 1 s per step. Powder samples were prepared in optical glass holders with 0.5 mm deep notches. All scans underwent normalization. For system calibration, the diffractometer was aligned using a NIST 1976b corundum standard to verify and adjust the instrumental parameters.^25^

Raman spectroscopy was performed by using a Renishaw inVia Raman microscope equipped with a 532 nm Nd:YAG laser. Measurements were conducted with a 0.75 numerical aperture objective, maintaining a laser power of 0.05 mW at the sample to minimize thermal effects. For statistical reliability, five spectra per sample were collected at different locations, each with an integration time of 30 s (5 accumulations). Sample powder was placed on a glass slide for conducting the measurements, and the measured spectra were cosmic-ray-corrected and normalized (0–1 range). The system was calibrated with a silicon standard (520.5 cm^–1^ peak) before and after each measurement session to ensure wavelength accuracy.^26^

Scanning electron microscopy (SEM) and energy-dispersive X-ray spectroscopy (EDX) were performed by using a ZEISS GEMINI 500 microscope (Oxford Instruments EDX detector). Imaging was performed using a 1 kV acceleration voltage to optimize surface morphology resolution, while EDX measurements utilized 15 kV to ensure sufficient X-ray excitation. Samples were analyzed before and after electrochemical testing to track compositional and morphological changes. Prior to analysis, specimens were mounted on aluminum stubs using double-sided copper tape to ensure electrical conductivity. For statistically robust EDX results, 20 random points per sample were analyzed, and the average elemental composition was calculated.

For characterizing the sample morphology and chemical composition by scanning transmission electron microscopy (STEM), a JEOL JEM-F200 transmission electron microscope operating at 200 kV was used. The microscope was equipped with a cold field emission electron source and a large, windowless JEOL Centurio EDX (Energy Dispersive X-ray emission) detector (100 mm^2^, solid angle of 0.97 sr, and energy resolution below 133 eV@MnKα), a CEOS CEFID energy filter, and two TVIPS XF416 CMOS cameras (pre- and postfilter). High-angle annular dark-field (HAADF) images, providing Z-contrast and EDX intensity maps, were obtained by using a beam current of 0.1 nA and a beam diameter of 0.16 nm. Sphere diameters, wall thicknesses, and iron species particle sizes were measured by using ImageJ software.

Furthermore, cryogenic transmission electron microscopy/electron energy loss spectroscopy (Cryo-TEM/EELS) and cryo-scanning transmission electron microscopy/electron energy loss spectroscopy were used to investigate the elemental distribution of a post-mortem electrode. The sample was maintained at a temperature of –170 °C in a GATAN Elsa cryo-TEM transfer holder.

Thermogravimetric analysis (TGA) was conducted by using a Netzsch TG 209 Libra thermobalance. Under controlled oxidative conditions (synthetic air with argon protective gas), mass changes were recorded during thermal ramping (10 °C min^–1^) up to 900 °C. The powder was loaded into the Al_2_O_3_ crucibles.

Elemental analysis (CHNS-O) was conducted using a Vario Micro Cube system (Elementar). Samples were weighed in tin boats with a consistent addition of WO_3_ and compressed to exclude air. Combustion was carried out in a combustion tube maintained at 1150 °C, while the reduction zone was maintained at 850 °C. The instrument calibration was performed by repeatedly analyzing sulfanilamide standards. A rapid OXY Cube analyzer (Elementar) was used to determine the oxygen content. Samples were weighed into silver boats, compressed to exclude air, and subjected to pyrolysis at 1450 °C. Calibration for oxygen analysis was achieved through multiple measurements of benzoic acid standards.

Pore size distribution (PSD) and the specific surface area (SSA) were determined by nitrogen sorption isotherms recorded at −196 °C using an Autosorb 6100 instrument (Anton Paar GmbH). Before the measurements, samples were degassed under vacuum at 180 °C for 24 h. SSA values and PSD analysis were calculated using the 2D nonlinear density functional theory (2D-NLDFT) for heterogeneous surfaces.^27^

X-ray photoelectron spectroscopy (XPS) measurements were conducted by using an Axis Supra spectrometer (Kratos Analytical). With wide-scan elemental spectra and high-resolution measured scans, data were collected using Al–Kα radiation at 225 W with pass energies of 160, 80, and 10 eV. Depth profiling was performed by alternating between Argon (Ar) cluster ion etching and XPS measurements to analyze the sample’s composition as a function of depth. A sequence of ion gun etch cycles was conducted, each followed by XPS acquisition to examine the newly exposed surface. The etching was carried out using an Ar GCIS (Minibeam 6, Kratos Analytical) operating at a cluster size of Ar_1000_^+^ with an impact energy of 10 keV over an area of 0.5 × 0.5 mm^2^. Two etch cycles were performed, each lasting 18,000 s, resulting in a total etch time of 36,000 s. Data processing and analysis were performed by using CasaXPS software (Casa Software, version 2.3.15).

Analysis of colloidal template solutions (PS) was performed using dynamic light scattering on a Malvern Zetasizer instrument with a light backscattering angle of 173°. One measurement consisted of 3 × 30 separate submeasurements.

### 2.3 Electrode Preparation

Electrochemical characterization was conducted by using working electrodes fabricated without conductive carbon additives. For the electrodes, a composition consisting of 90 mass % synthesized active material (CS_Fe_Low, CS_Fe_Med, CS_Fe_High) and 10 mass % polyvinylidene fluoride (PVdF, Alfa Aesar) as the binder was prepared. The binder was dissolved in *N*-methyl-2-pyrrolidone (NMP, 99.9% purity, Sigma-Aldrich) to form a slurry. For electrode preparation, we ground the dry active material in a mortar to ensure a uniform particle size. The ground powder was mixed using a SpeedMixer DAC 150 SP (Hauschild) instrument at 1000 rpm for 5 min. The NMP was then added to create a viscous paste, which underwent sequential mixing at 1500 rpm for 5 min and 2500 rpm for 10 min. A 10 mass % PVdF solution in NMP was subsequently incorporated into the paste, followed by additional mixing at 800 rpm for 10 min. The final slurry was homogenized by continuous stirring with a magnetic stirrer for 12 h.

The prepared slurry was applied to a 25 μm thick copper foil (MTI) by using a doctor blade (ZUA 2000.80 Proceq universal applicator) set to a 200 μm gap. The coated electrodes were dried in a fume hood under ambient conditions before undergoing vacuum drying at 110 °C for 12 h to remove residual NMP. The final electrode sheets had a thickness of 30–40 μm and an active material loading of approximately 1.8 ± 0.3 mg cm^–2^. Including the copper current collector, the total electrode mass was measured at 25.5 ± 1 mg.

### 2.4 Electrochemical Characterization

For electrochemical benchmarking, working electrodes were punched into 12 mm disks (1.131 cm^2^ area) using a press punch from EL-CELL. These disks were incorporated into a CR2032 coin-cell (MTI) configuration as the working electrode. Prior to assembly, all of the cell components were vacuum-dried at 120 °C for 12 h. The assembly process was carried out in an argon-filled glovebox (MBraun) with oxygen and water vapor concentrations maintained below 0.1 ppm. Lithium disks (11 mm diameter) were used as both the counter and reference electrodes, while an 18 mm diameter vacuum-dried glass-fiber disc (Whatman GF/F) served as a separator. The electrolyte consisted of a 1 M solution of lithium hexafluorophosphate (LiPF_6_) in a 1:1 volumetric mixture of ethylene carbonate and dimethyl carbonate (EC/DMC), sourced from Sigma-Aldrich. Approximately 150 μL of the electrolyte was added to each cell to ensure proper wetting and ion transport.

All electrochemical measurements were performed under temperature-controlled conditions (+25.0 ± 1.0 °C) using a Binder climate chamber to ensure environmental stability. Cyclic voltammetry (CV) experiments were conducted using a Biologic VMP-300 multichannel potentiostat/galvanostat and a BCS-810 battery cycler (Bio-Logic) equipped with the EC-Lab/BT-Lab software. Potential scans ranged from 0.01 to 3.00 V vs. Li^+^/Li at a scan rate of 0.1 mV s^–1^. For further kinetic analysis employing various scan rates, we employed 0.10 mV s^–1^, 0.25 mV s^–1^, 0.50 mV s^–1^, 0.70 mV s^–1^, 1.00 mV s^–1^, 2.50 mV s^–1^, 5.00 mV s^–1^, 7.50 mV s^–1^, and 10.00 mV s^–1^. Galvanostatic charge/discharge cycling with potential limitation (GCPL) experiments were also conducted using a Bio-Logic battery cycler to evaluate the electrodes’ rate performance. These tests were performed in the same potential window (0.01–3.00 V vs. Li^+^/Li) across different specific currents (0.05 A g^–1^, 0.10 A g^–1^, 0.20 A g^–1^, 0.50 A g^–1^, 1.00 A g^–1^, 2.00 A g^–1^, 4.00 A g^–1^, 8.00 A g^–1^) and returning to 0.10 A g^–1^. All currents and capacities were normalized to the active mass of the electrode, which consisted of 90 mass % of CS_Fe_Low, CS_Fe_Med, or CS_Fe_High. At least three cells of each material were tested for each experimental condition to ensure reproducibility, and all reported results were based on consistent data from individual cells. Long-term cycling stability measurements were made with a charge/discharge current of 0.10 A g^–1^.

For post-mortem electrode analysis, the cells were first stabilized at a potential of 3.00 V for 12 h to ensure complete de-lithiation. Disassembly of the cells was carried out in a glovebox under controlled conditions to avoid exposure to ambient air or moisture, which could alter the electrode’s properties. The electrodes were then washed thoroughly with 5 mL of dimethyl carbonate (DMC; ≥99% purity, Sigma-Aldrich) to remove residual electrolyte salts. Subsequently, the washed electrodes were vacuum-dried at room temperature to eliminate any remaining solvent, ensuring their readiness for subsequent analytical characterization.

## 3 Results and Discussion

### 3.1 Synthesis of Iron-Loaded Hybrid Carbon Spherogels

We recently reported that homogeneous hybrid carbon spherogels (titania-loaded) can be synthesized by incorporating metal-organic precursors into our robust synthesis pathway, generating monolithic carbon spherogels.^28^ In this work, we apply this knowledge to utilize iron lactate and encapsulate it into hybrid carbon spherogels through a polystyrene sphere-templated resorcinol-formaldehyde (RF) sol-gel process. Figure 1 depicts a schematic overview of the material synthesis and processing. The synthesis starts by preparing an aqueous solution containing 9 mass % monodispersed PS spheres with an average size of 204 ± 4 nm (Supporting Information, Figure S1) and resorcinol. In the next step, adding the desired iron lactate aqueous solutions enables the loading of iron precursors (iron lactate) with various amounts, leading to different final loadings named CS_Fe_Low, CS_Fe_Med, and CS_Fe_High. Negatively charged PS spheres (due to the presence of sulfate groups on the outer surface) are preferential sites for the formation and arrangement of iron^2+^ species, which form and arrange around the PS spheres. Subsequently, methylation occurs through the reaction between resorcinol and formaldehyde in the presence of the sodium carbonate catalyst, forming hydroxymethyl groups, followed by polycondensation.^29^ Next, oligomeric species grow during gelation at 80 °C through condensation reactions between resorcinol and formaldehyde. This allows the coating of PS spheres decorated by iron lactate molecules, forming an interconnected 3D RF/iron-lactate-PS gel network. Afterward, supercritical drying with CO_2_ resulted in dried monolithic RF/iron-PS gels with minimal shrinkage. Finally, heat treatment of the obtained monoliths at 800 °C under an argon atmosphere with a low heating rate of 1 °C min^–1^ transfers monolithic RF/iron-PS gels to the iron-loaded carbon spherogels (concomitant template removal, carbonization, and iron lactate decomposition). As a result, uniform and microporous nanostructured carbon shells are formed through the carbonization of RF resin, serving as the carbon source. Interior cavities arise from the removal of the PS cores, while iron nanoparticles are generated through the decomposition of iron lactate, followed by subsequent reduction.

**Figure 1:**
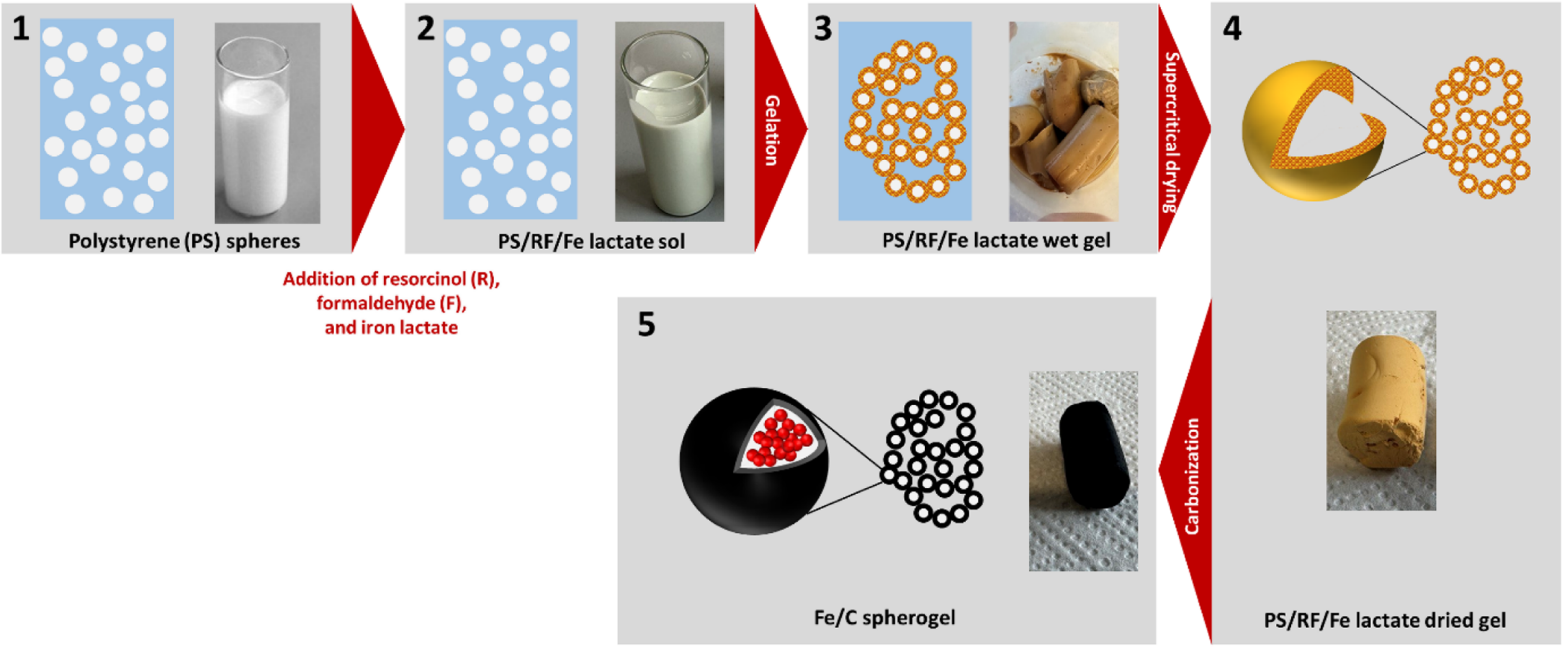
Schematic drawing of the synthesis procedure of iron-loaded carbon spherogels.

### 3.2 Structural and Chemical Material Characterization

The synthesized iron-species-loaded carbon spherogels underwent detailed chemical characterization. The scanning electron micrographs and scanning transmission electron micrographs (Figure 2 and Supporting Information, Figure S2) provide insights into the morphology of the iron-loaded spherogels. All samples show a homogeneous 3D structure composed of highly uniform interconnected carbon spheres, loaded and decorated with Fe nanoparticles, confirming the successful synthesis process with optimized parameters for the formation of RF/iron-PS gels. CS_Fe_Low (10% iron lactate precursor) displays a compact structure with uniformly distributed spherical carbon spheres. Additionally, small particles visible outside the carbon shells can be attributed to partially agglomerated iron species.

**Figure 2:**
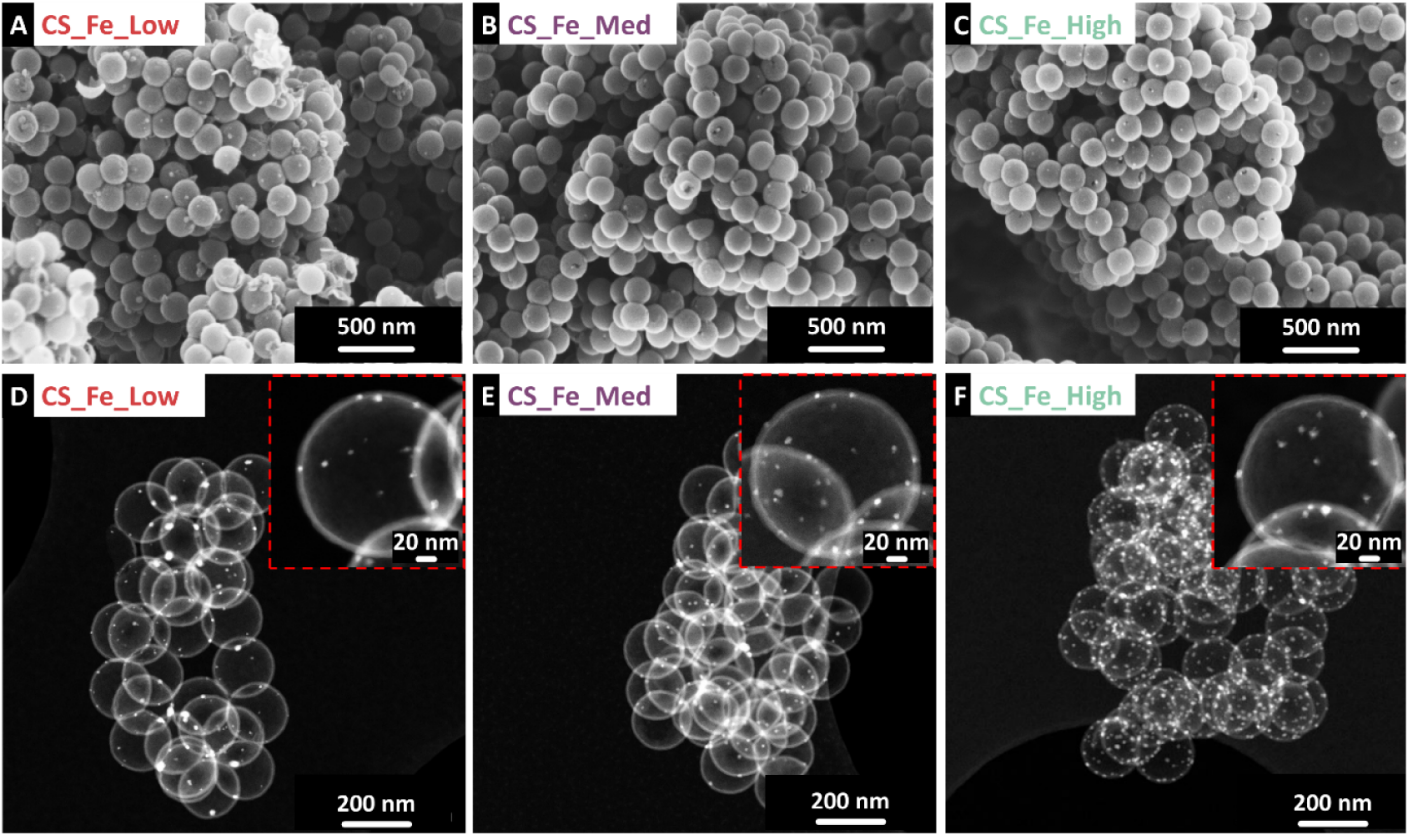
Scanning electron micrograph of (A) CS_Fe_Low, (B) CS_Fe_Med, (C) CS_Fe_High. Scanning transmission electron micrographs of (D) CS_Fe_Low, (E) CS_Fe_Med, and (F) CS_Fe_High.

In contrast, CS_Fe_Med (20% iron lactate precursor) demonstrates a more homogeneous sphere morphology for the iron species. At the same time, the carbon spherogel network is similar, suggesting an optimized balance between the iron content in the host and structural integrity. CS_Fe_High (30% iron lactate precursor) retains a well-defined, uniform spherical morphology, indicating that higher iron loading does not compromise the structural stability of the carbon spherogels. This structural robustness, combined with the conductivity and active properties of iron, highlights the potential of iron-loaded carbon spherogels as advanced electrode materials for LIBs.

Scanning transmission electron micrograph observations in Figure 2D-F, along with the corresponding size distribution histograms in Supporting Information, Figure S3, reveal that iron-based nanoparticles, with an overall particle size range of 7 to 35 nm, are homogeneously encapsulated within interconnected carbon hollow spheres. The low-, medium-, and high-iron samples (CS_Fe_Low, CS_Fe_Med, and CS_Fe_High) show iron-based nanoparticles with average diameters of 16 ± 7, 12 ± 3, and 15 ± 6 nm, respectively.

Scanning transmission electron micrographs (Figure 2D-F) show that iron-based nanoparticles with a 10 nm-30 nm diameter range are homogeneously encapsulated into interconnected carbon hollow spheres. Carbon spherogels in all samples have an interior diameter of 170 ± 4 nm, indicating the uniform, monomodal use of a 9 mass % PS template with an average size of 204 ± 1 nm during synthesis. The difference between the interior diameter and PS size is due to the slight shrinkage typically induced by carbonization. Elemental analysis by scanning transmission electron microscopy EDX for the elements Fe, C, and O (Supporting Information, Figure S4) further verifies the formation of carbon spherogels with highly homogeneous structures in terms of the inner/outer diameter and wall thickness, embedded with iron-based nanoparticles for the low, medium, and high iron-loaded samples.

Raman spectra of the three synthesized hybrid carbon spherogels with different contents of iron loading, ranging from 10% to 30% iron lactate precursor (Figure 3A) show the characteristic D-band and G-band of the carbon framework, with overall minimal variations in intensity and peak position, reflecting similarities in defect density and graphitization. Small differences arise from the increasing iron loading, which can influence the structural characteristics of the carbon matrix. The X-ray diffractograms shown in Figure 3B display, in addition to the broad carbon-characterizing signal around 20° 2θ for all samples, reflections corresponding to elemental iron (PDF#03-065-4899), with variations in peak intensity and sharpness that indicate differences in crystallinity and distribution of the iron particles among the samples. Higher iron loading (CS_Fe_High) appears to promote a more pronounced crystalline phase, indicating a higher iron loading of the carbon spherogels and enhanced accessibility.

**Figure 3:**
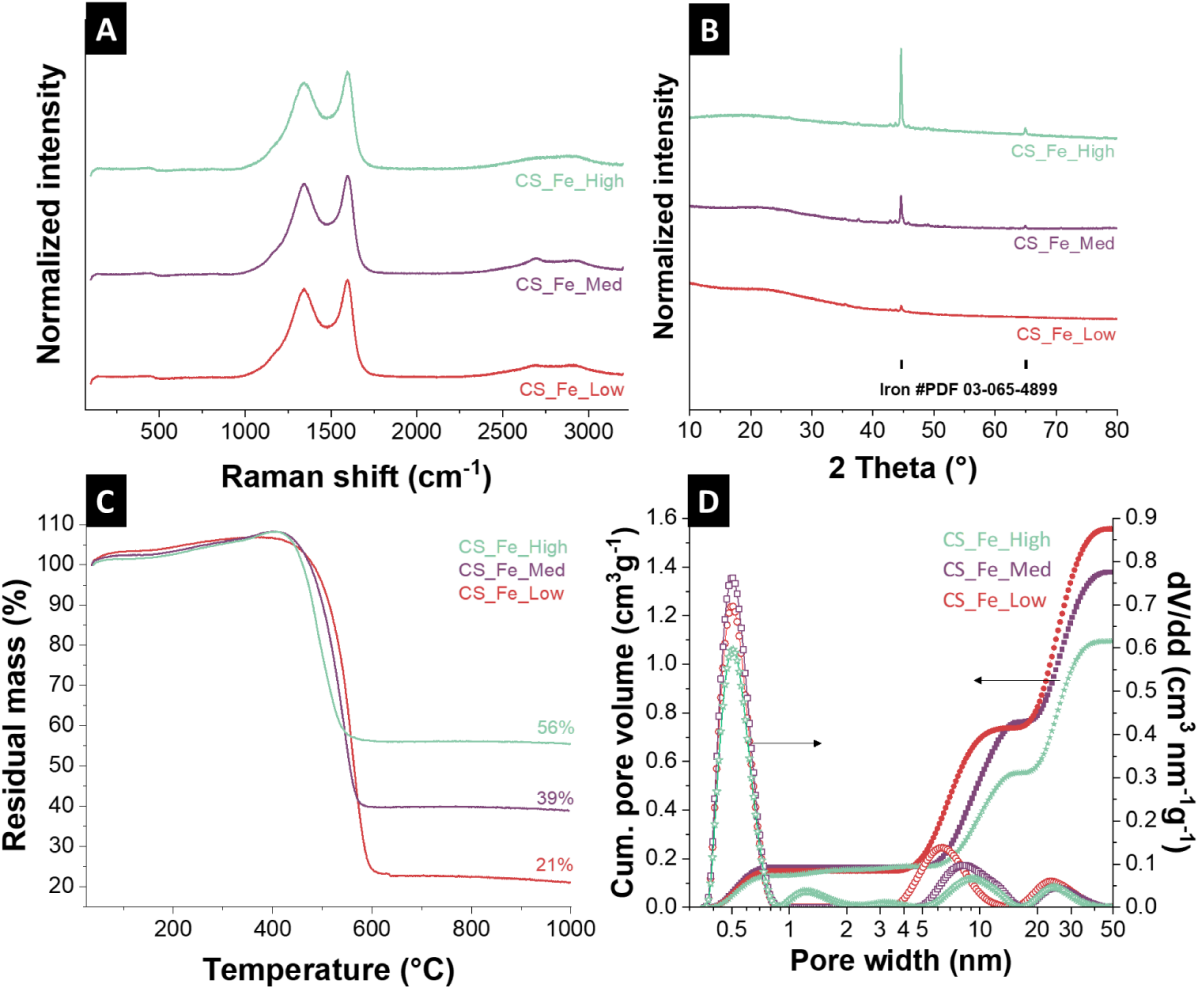
Chemical characterization of the different iron-loaded carbon spherogels (CS_Fe_Low, CS_Fe_Med, CS_Fe_High) showing (A) Raman spectra, (B) X-ray diffractograms using Cu–Kα radiation, (C) thermogravimetric analysis, and (D) cumulative pore size distributions (left axis) and differential pore size distributions (right axis) determined using 2D-NLDFT.

To better understand the successful loading of carbon spherogels, elemental analysis (CHNS-O) and thermogravimetric analysis (TGA) were conducted. The data summarized in Figure 3C and Table 1 show a clear trend of decreasing carbon and hydrogen content as the iron lactate percentage present during synthesis increases in the samples. Specifically, as the iron lactate concentration rises from 10% (CS_Fe_Low) to 30% (CS_Fe_High), the carbon mass percentage drops from 80.19 mass % to 59.26 mass %, while the hydrogen mass percentage slightly decreases from 0.666% to 0.269%, and the oxygen percentage reduces from 8.091 mass % to 6.047 mass %. This suggests that the addition of iron lactate to the material might partially replace carbon and hydrogen, likely due to the incorporation of iron into the structure. The absence of nitrogen and sulfur indicates the high purity of the iron-loaded carbon spherogels, free from trace impurities introduced during synthesis. To estimate the mass fraction of metallic iron (Fe^0^) within the samples after TGA, a complete oxidation of iron to hematite (Fe_2_O_3_) was assumed. Based on the observed mass increase during heating in an oxidative atmosphere, the final mass corresponds to the formation of Fe_2_O_3_. Using stoichiometric considerations and the molar mass ratio between Fe and Fe_2_O_3_, the theoretical mass fraction of elemental iron in each sample was calculated. The calculations were performed for samples obtained via 10 mass %, 20 mass %, and 30 mass % iron lactate precursor (CS_Fe_Low, CS_Fe_Med, and CS_Fe_High). The resulting estimated Fe^0^ content in the final material was 14.7 mass % for CS_Fe_Low, 27.3 mass % for CS_Fe_Med, and 39.2 mass % for CS_Fe_High. These values represent the maximum theoretical metallic iron content, assuming complete conversion to Fe_2_O_3_ and no loss of iron-containing species during synthesis or measurement. All of the initially present iron is considered as Fe^0^.

**Table 1:** Elemental analysis of different iron-loaded carbon spherogels by CHNS-O analysis in mass % and calculated elemental iron values obtained from TGA analysis considering the reaction to Fe_2_O_3_ ^a^

	Iron lactate precursor amount (mass %)	Carbon (mass %)	Hydrogen (mass %)	Nitrogen (mass %)	Sulfur (mass %)	Oxygen (mass %)	Iron (mass %)
**CS_Fe_Low**	10	80.19 ± 2.36	0.67 ± 0.03	/	/	8.09 ± 0.36	14.69
**CS_Fe_Med**	20	71.40 ± 4.77	0.54 ± 0.11	/	/	6.74 ± 0.29	27.27
**CS_Fe_High**	30	59.26 ± 4.32	0.27 ± 0.05	/	/	6.05 ± 0.33	39.16

a Values below the detection limit are noted as “/”.

The pore structure and specific surface area of the samples were characterized and calculated using nitrogen adsorption measurements (Figure 3D and Supporting Information, Figure S5). The nitrogen sorption isotherms for all samples (Supporting Information, Figure S5) indicate a combination of type I and special features of the hollow sphere character: at low relative pressures (p/p_0_ ≤ 0.1) the sharp initial uptake indicates nitrogen adsorption by micropores in the sphere walls, followed by a plateau (p/p_0_ = 0.1–0.9), typical for microporous carbons (diameter below 2 nm).^30^ This observation agrees with the micropore size distribution centered below 1 nm in the DFT-derived pore size distribution (Figure 3D). At high relative pressures (p/p_0_ > 0.9), a second, significant nitrogen uptake takes place by filling the interior voids of the hollow spheres. This process is strongly dependent on the measurement parameters (data points and equilibration time) and is, ultimately, not completed by a plateau feature. Thus, due to the combined effect of iron nanoparticles and insufficient interior filling, the typical H2a hysteresis (caused by cavitation) is hindered and only partially present.^31^ We observe this hindering effect more pronounced with increasing Fe loading (Supporting Information, Figure S5).^8,32^ The SSA and total pore volume for the samples were calculated by employing the 2D-NLDFT method (heterogeneous surface model). The CS_Fe_Low sample exhibits the highest SSA (807 m^2^ g^–1^), followed by CS_Fe_Med (802 m^2^ g^–1^) and CS_Fe_High (662 m^2^ g^–1^), indicating a decreasing trend with increasing iron content. A similar trend is observed for the total pore volume (up to 30 nm): as shown in Figure 3D, the CS_Fe_Low sample exhibits the highest pore volume (1.56 cm^3^ g^–1^) compared to CS_Fe_Med (1.37 cm^3^ g^–1^) and CS_Fe_High (1.10 cm^3^ g^–1^).

### 3.3 Electrochemical Performance of Iron-Loaded Hybrid Carbon Spherogels

To characterize the electrochemical performance of the iron-loaded hybrid carbon spherogels with different iron contents, Figure 4A,C,E and Supporting Information, Figure S6 show cyclic voltammograms (at different scan rates) for the three samples (10 mass %, 20 mass %, and 30 mass % iron lactate precursor). The observed bands characterize the redox behavior of the material during lithium-ion insertion and extraction. All samples exhibit a distinct reduction peak at approximately 0.63 V vs. Li^+^/Li, which corresponds to the formation of a solid electrolyte interphase (SEI) and the complete reduction of Fe species (Fe^3+^ and Fe^2+^) to Fe^0^.^33^ In subsequent cycles, this peak diminishes and evolves into a new peak at around 0.81 V vs. Li^+^/Li, attributed to the reversible lithium insertion and the complete reduction of Fe^2+^O to Fe^0^.^22,34^ The broad and extended oxidation peak at approximately 1.3 V vs. Li^+^/Li corresponds to the reversible oxidation of Fe^0^ to Fe^3+^. Over five cycles, the redox peaks stabilize, indicating reversible electrochemical behavior. The peak intensities and positions remain largely unchanged across all samples, suggesting that the iron lactate precursor content has a minimal influence on reaction kinetics and capacity, while confirming the electrochemical addressability of redox-active species in each sample.

**Figure 4:**
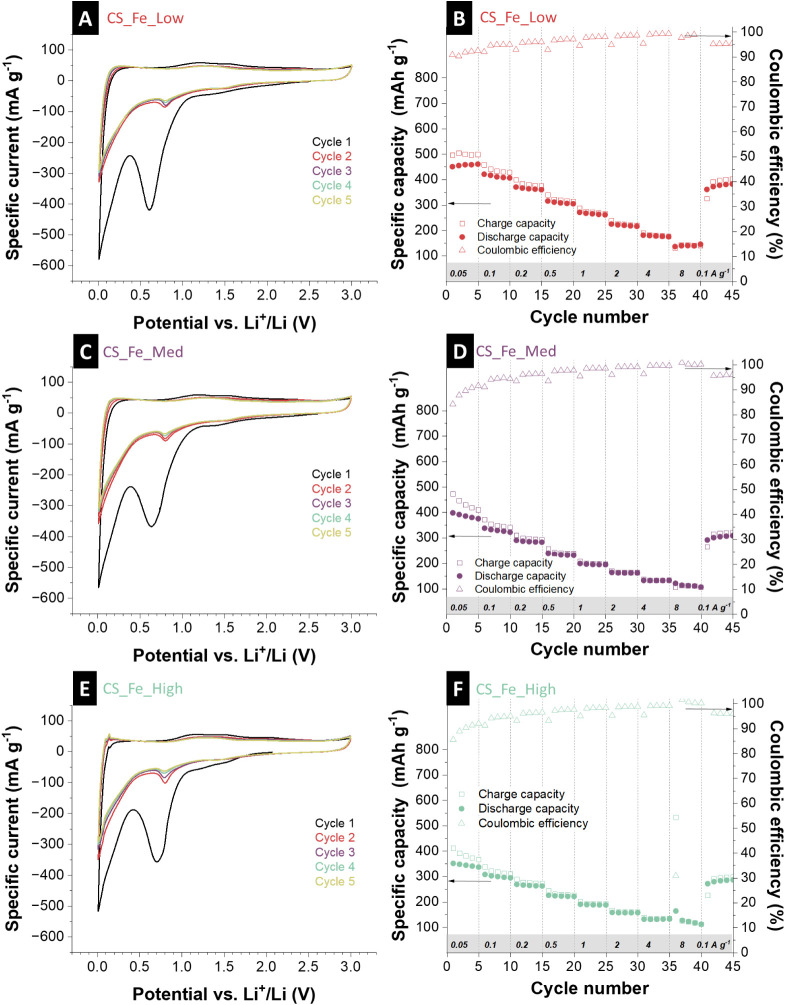
Electrochemical characterization of hybrid iron-loaded carbon spherogels showing cyclic voltammograms at a scan rate of 0.10 mV s^–1^ for 5 cycles for (A) CS_Fe_Low, (C) CS_Fe_Med, and (E) CS_Fe_High. Rate handling ability during galvanostatic charge/discharge cycling at different rates, along with the values for the Coulombic efficiency, is shown for (B) CS_Fe_Low, (D) CS_Fe_Med, and (F) CS_Fe_High.

Kinetic studies were conducted to provide a more detailed characterization of the material’s potential pseudocapacitive properties, with a specific focus on analyzing how the current signal changes with different scan rates (Supporting Information, Figure S7). The fundamental relationship between the measured current (*i*) and the scan rate (*v*) was described using the power-law equation *i* = *av* ^*b*^, from which the fitting parameters *a* and *b* were derived. In this model, the *b*-value is a critical diagnostic parameter: an ideal value of 0.5 is representative of a semi-infinite diffusion-limited process, which is typical for battery-type electrode materials. Conversely, a *b*-value of 1.0 signifies a surface-limited charge storage mechanism, such as that found in capacitive systems or rapid ion electrosorption.^35^

A more comprehensive analysis of the *b*-values for the various iron-loaded samples, calculated across a scan rate range from 0.10 mV s^–1^ to 1.00 mV s^–1^, is provided in Supporting Information, Figure S6. The data reveal that the *b*-values for all of the synthesized samples are consistent with one another. Specifically, at the potential associated with a slight redox feature, the calculated *b*-value was approximately 0.77 for every sample. These elevated *b*-values, being significantly greater than 0.5, strongly suggest that the charge storage process at this potential is not purely diffusion-limited but instead has a substantial surface-controlled component.

This trend toward surface-limited dominance becomes even more pronounced at a potential of 0.50 V vs. Li^+^/Li. At this point, the *b*-values for all samples converge at 0.98, which indicates an electrochemical response that is almost entirely governed by pseudocapacitive behavior and a surface-limited process. The validity of this conclusion is further reinforced by examining the lithiation and de-lithiation curves, which exhibit a nearly linear relationship between the charge stored and the cell voltage, particularly after the initial formation cycles. Such linearity is a recognized hallmark of surface-limited processes and provides independent, corroborating evidence for the findings from the *b*-value analysis, thereby highlighting the unique electrochemical characteristics of the investigated samples. In contrast, at a higher potential of 2.75 V vs. Li^+^/Li, the calculated *b*-values fall within a narrower range of 0.60 to 0.63. These lower values suggest a definitive shift in the reaction mechanism, pointing toward a process that is rather battery-like in nature, likely involving the diffusion-limited reaction of lithium ions with the iron species present in the electrode material.

The rate handling capability (Figure 4B,D,F) includes galvanostatic charge and discharge capacities, along with the respective Coulombic efficiency at specific currents ranging from 0.05 A g^–1^ to 8.00 A g^–1^. The CS_Fe_Low sample exhibits the highest de-lithiation capacity throughout the rate test with an initial de-lithiation capacity of 451 mAh g^–1^, while CS_Fe_Med and CS_Fe_High perform very similar but slightly lower capacity (initial de-lithiation capacities of 398 mAh g^–1^ and 352 mAh g^–1^, respectively). For all samples, the initial capacity diminishes over time, particularly at higher specific currents, suggesting a trade-off between capacity and rate stability. The sample with medium iron content (CS_Fe_Med) displays a capacity retention of 96% when returning to a rate of 0.10 A g^–1^ with slightly reduced overall capacity compared with the CS_Fe_Low sample, which increases the initial capacity (115%). In comparison, the CS_Fe_High sample demonstrates the most stable cycling performance (103%), albeit with the lowest initial capacity. These results suggest that increasing iron lactate precursor content enhances structural integrity and long-term reversibility but reduces the initial capacity. Furthermore, the increase in capacity during cycling and the capacity retention exceeding 100% indicate an activation process of electrochemically active species in the electrode material.

Overall, the CS_Fe_Low material is suitable for applications that prioritize high initial capacity, while the CS_Fe_High material is better suited for achieving a balanced capacity increase, albeit with a capacity loss beyond 300 cycles. The CS_Fe_Med sample represents a balance between capacity and stability, offering intermediate performance.

The obtained reduction and oxidation peaks from cyclic voltammetry agree with the galvanostatic discharge and charge plateaus, indicating the materials’ lithium-ion insertion and extraction processes tested at 0.10 A g^–1^ in a voltage range between 0.01 V vs. Li^+^/Li and 3.00 V vs. Li^+^/Li (Figure 5A,C,E). The CS_Fe_Low sample shows an initial capacity of about 612 mAh g^–1^. Still, its voltage profile changes to significantly higher capacities (1190 mAh g^–1^) with cycling, reflecting a substantial capacity increase (194%) by the 300^th^ cycle. In contrast, the CS_Fe_Med sample maintains a more stable voltage profile with a moderate capacity increase (148% after 300 cycles). In comparison, the CS_Fe_High sample demonstrates the highest stability, showing minimal changes (102%) in its voltage profile, even after 200 cycles.

**Figure 5:**
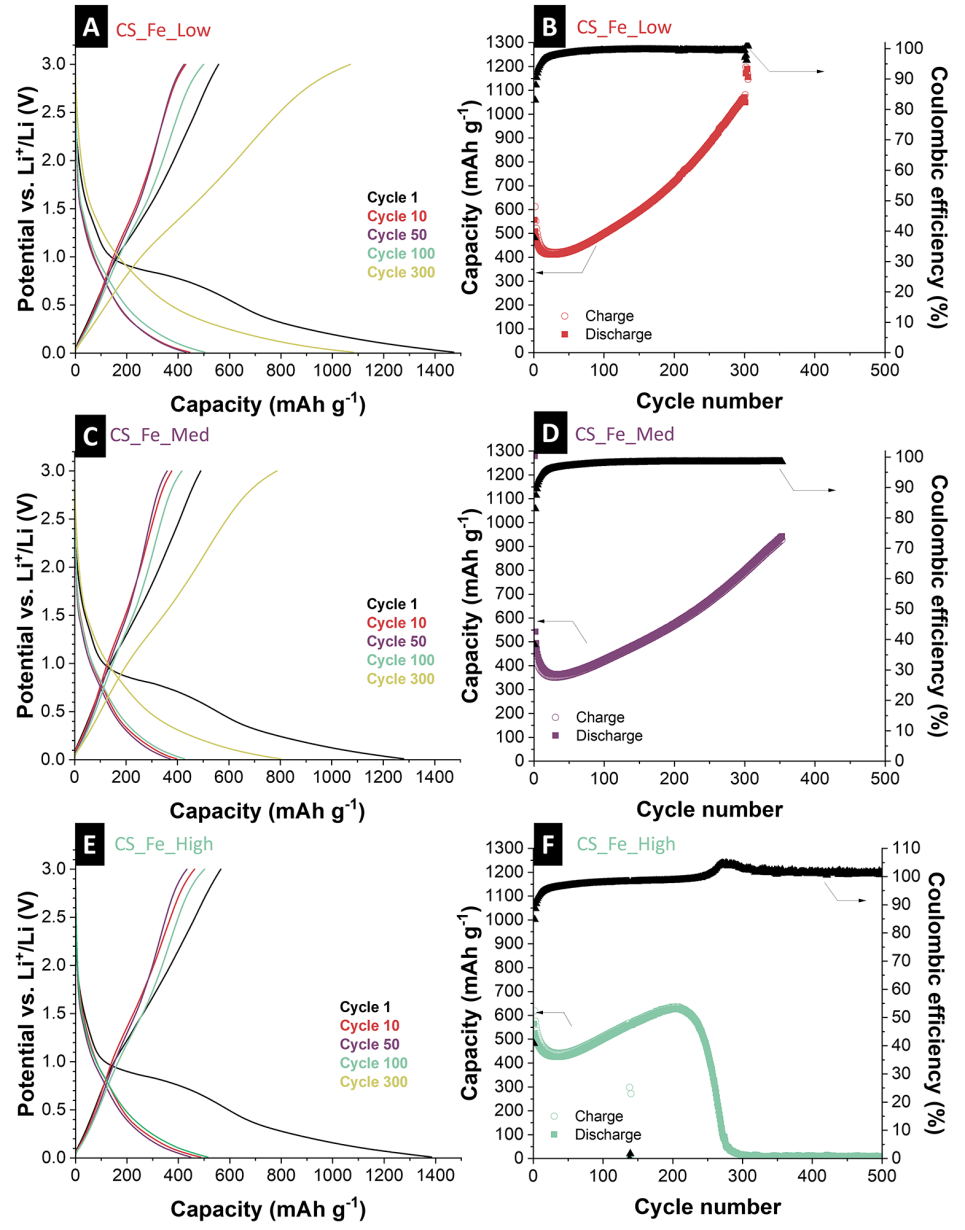
Electrochemical characterization of hybrid iron-loaded carbon spherogels showing galvanostatic lithiation and de-lithiation profiles at an applied specific current of 0.10 A g^–1^ between 0.01 and 3.00 V vs. Li^+^/Li for (A) CS_Fe_Low, (C) CS_Fe_Med, and (E) CS_Fe_High galvanostatic charge/discharge cycling performance, electrochemical stability, and corresponding Coulombic efficiency values for (B) CS_Fe_Low, (D) CS_Fe_Med, and (F) CS_Fe_High.

Figure 5B,D,F summarizes the cycling performance and Coulombic efficiency of CS_Fe_Low, CS_Fe_Med, and CS_Fe_High over extended cycling (350 cycles). The CS_Fe_Low electrode demonstrates high cycling performance, with an initial specific discharge capacity of approximately 612 mAh g^–1^. The capacity continues to increase after the initial 20 cycles during prolonged cycling, reaching 1190 mAh g^–1^ after 300 cycles. The initial capacity decrease of all iron-loaded carbon spherogels can be attributed to free iron species in the material, which are captured by the spherogels. Due to the free iron loading, the volume expansion during cycling cannot be effectively buffered by the carbon framework, leading to structural pulverization and a loss of electrochemical activity, which ultimately results in a decline in capacity.

In contrast, the gradual activation behavior observed for the optimized samples is accompanied by a consistently high Coulombic efficiency exceeding 99%, indicative of highly reversible lithiation and de-lithiation. The continuous increase in capacity over cycling can be attributed to the progressive activation of additional iron species embedded within the carbon spherogel matrix, which become electrochemically accessible over time. Furthermore, the initially metallic Fe^0^ species are slowly oxidized to iron-oxide-rich phases during repeated cycling, further contributing to enhanced electrochemical activity and improved capacity retention. The good cycling stability is attributed to the optimized Fe content and the robust carbon spherogel framework, which ensures efficient charge transport and mitigates mechanical degradation.

A similar trend, albeit with lower absolute capacity values, is observed for the CS_Fe_Med electrode. Starting from 542 mAh g^–1^, the capacity progressively rises to approximately 802 mAh g^–1^ after 300 cycles. The slightly reduced performance compared to that of CS_Fe_Low indicates that a moderate increase in iron loading preserves the advantageous structural features of the carbon spherogel, albeit at the cost of ion transport kinetics and structural flexibility. Additionally, the lower specific capacities suggest that not all of the incorporated iron species are electrochemically accessible.

In contrast, the CS_Fe_High electrode showed pronounced instability. Although the initial capacity is comparable (619 mAh g^–1^), a rapid capacity fading set in after 200 cycles, culminating in almost complete capacity loss beyond 300 cycles. A strong increase in the Coulombic efficiency parallels the collapse in performance. This degradation is likely caused by excessive iron loading, which impairs the mechanical stability of the electrode, promotes irreversible side reactions, and leads to active material pulverization during repeated cycling.

These results highlight the critical role of iron content in balancing structural stability, charge transport, and electrochemical reversibility in Fe-carbon hybrid electrodes. Specifically, a lower Fe loading (<20 mass %) appears to be optimal for harnessing the synergistic effects between the conductive carbon network and the active iron species, thereby enabling superior long-term performance in LIBs.

#### 3.3.1 Post-Mortem Analysis

To evaluate the mechanism of capacity development of the most stable sample (CS_Fe_Med), post-mortem analysis was conducted after 1 completed cycle, after 23 cycles (the lowest achieved capacity), and after 230 cycles (the highest achieved capacity). X-ray diffraction, Raman spectroscopy, and scanning transmission electron microscopy were applied, together with corresponding EDX maps, to assess structural and morphological changes at various cycling stages. The X-ray diffractograms in Figure 6A demonstrate the evolution of the crystalline structure compared to the as-synthesized material. The pristine material exhibits sharp and well-defined reflections, indicative of its initial phase. After one cycle, these reflections remain largely unchanged, suggesting minimal structural alterations. However, by 23 cycles, reflection broadening and reduced intensity become evident, signaling increased disorder and partial amorphization. After 230 cycles, the X-ray diffractogram reveals significant structural degradation, including the emergence of reflections attributed to metallic Fe and Cu (current collector) phases, indicating reduction processes that occur during extended cycling.

**Figure 6:**
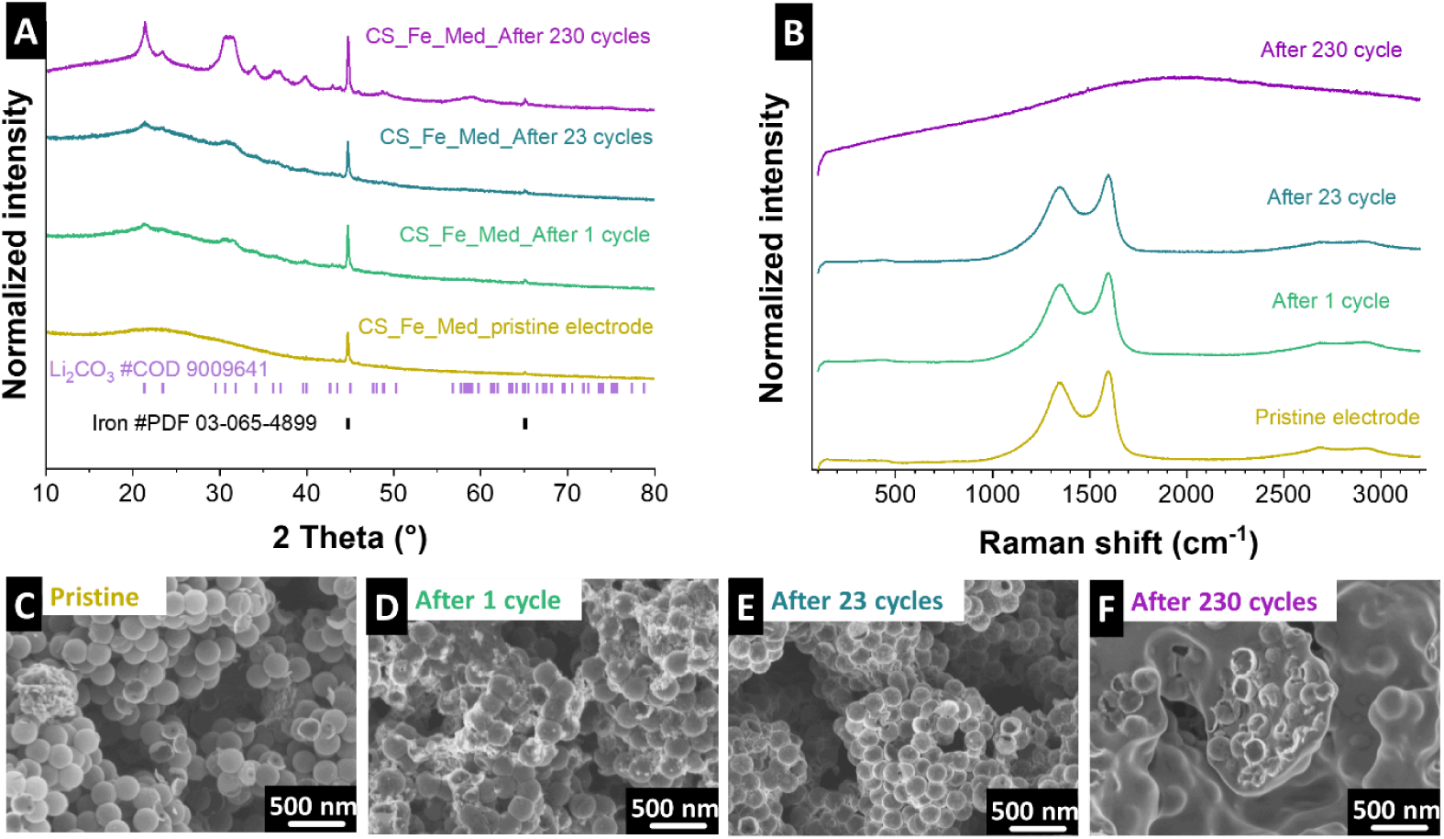
Post-mortem analysis showing (A) X-ray diffractograms of CS_Fe_Med pristine electrode, CS_Fe_Med after 1 completed cycle, CS_Fe_Med after 23 cycles, and CS_Fe_Med after 230 cycles. (B) Raman spectra of CS_Fe_Med pristine electrode, CS_Fe_Med after 1 completed cycle, CS_Fe_Med after 23 cycles, and CS_Fe_Med after 230 cycles. Scanning electron micrograph of the (C) CS_Fe_Med pristine electrode, (D) CS_Fe_Med after one completed cycle, (E) CS_Fe_Med after 23 cycles, and (F) CS_Fe_Med after 230 cycles.

The Raman spectra in Figure 6B further highlight the chemical changes in the material. The pristine sample shows distinct peaks corresponding to its well-ordered initial state. After one cycle, these peaks broaden slightly, reflecting early signs of structural reorganization. By 23 cycles, the broadening becomes more pronounced, pointing to the development of structural disorder. After 230 cycles, the spectrum flattens substantially, confirming extensive amorphization and loss of the original chemical structure or coating of the material.

The scanning electron micrographs in Figure 6C–F and Supporting Information, Figure S8 provide a detailed view of the material’s morphological evolution. The pristine material exhibits a uniform, well-dispersed, and highly porous carbon spherogel structure, which is essential for efficient lithium-ion transport. After one cycle, the overall morphology remains intact, with minor surface roughening likely due to the initial formation of the SEI. By 23 cycles, significant agglomeration of particles and surface irregularities are observed, indicating progressive structural changes. This may be attributed to a resistive and uneven SEI layer, as well as localized collapse of the porous structure, which hinders Li-ion diffusion. Additional scanning transmission electron microscopy micrographs, taken after 230 cycles at higher magnifications (Supporting Information, Figure S9), reveal that the carbon spherogels remain intact even after extensive cycling, forming a porous texture.

Cryo-STEM-EELS intensity maps and cryo-TEM-EELS spectra of the CS_Fe_Med electrode after 230 cycles, collected from two distinct regions, are shown in Figure 7. The cryo-STEM-HAADF micrograph of the electrode and corresponding STEM-EELS intensity maps in Figure 7A show a carbon sphere (C-rich zone, indicated with a yellow arrow) and a thick SEI layer, including Li, C, O, and F, which are key components previously reported in LIB studies.^36^ Figure 7B, F shows the Li–K edge in the EELS spectra, acquired in the regions shown in Figure 7A, E. Two distinct peaks, a sharp peak with a maximum at ∼62 eV and a broader one at ∼70 eV, with an energy separation of approximately 7.7 eV, suggest that LiF is the dominant Li-based inorganic compound in the SEI layer, in agreement with previous reports.^37^ However, the C–K edge and O–K edge in the EELS spectra of both areas (Figures 7C,G) indicate the presence of some Li_2_CO_3_ and Li_2_O due to the decomposition of the electrolyte.^38^ F–K and Fe–L edges in the EELS spectra (Figures 7D,H) verify the presence of elements F and Fe. The Fe-related peaks (Fe–L_2_ and Fe–L_3_) in Figure 7H are more evident due to the smaller scan area, where one Fe particle is located (bright area in the inset of the STEM-HAADF micrograph, Figure 7E).

**Figure 7:**
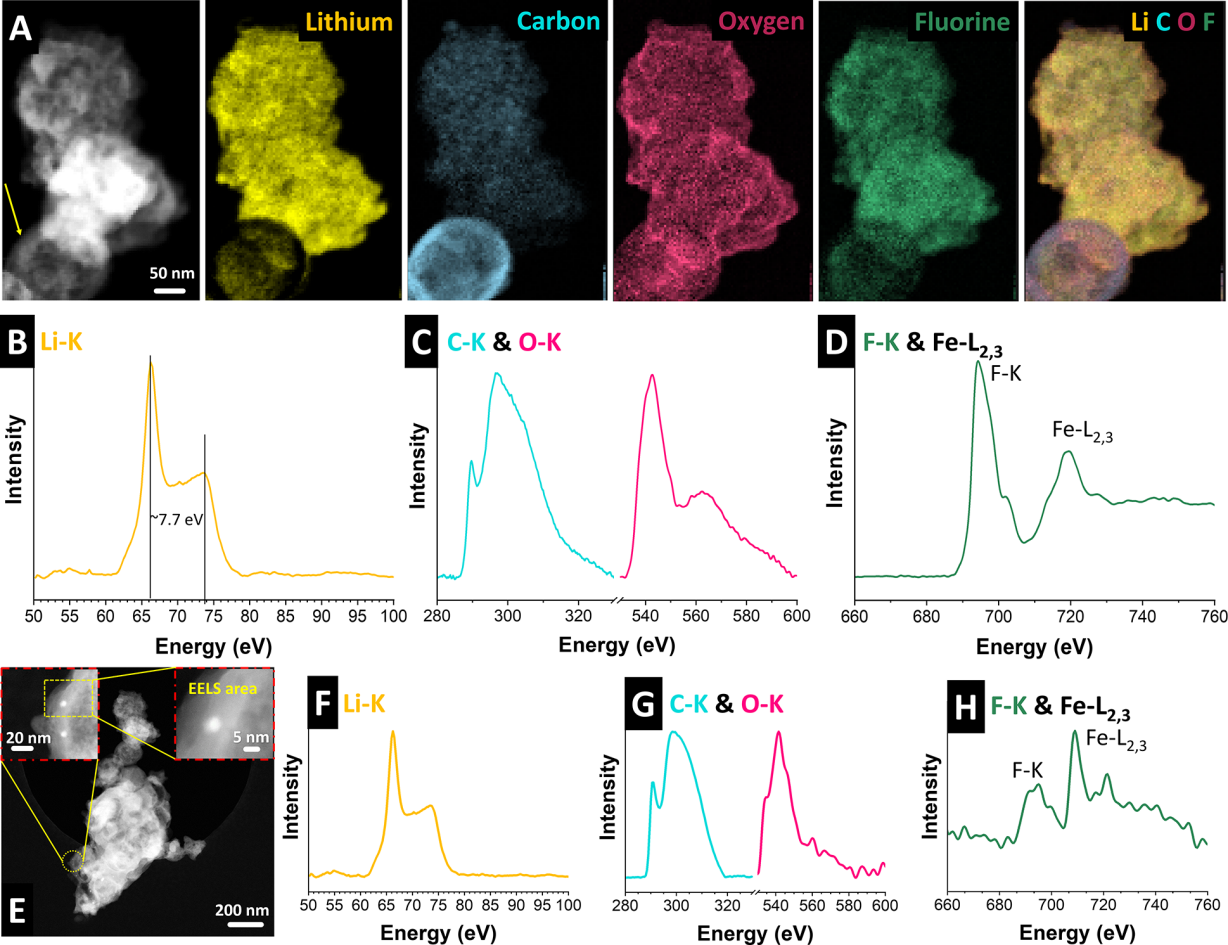
Post-mortem cryo-scanning transmission electron microscopy and cryo-TEM-electron energy loss spectroscopy analysis of the CS_Fe_Med electrode after 230 cycles. (A) Scanning transmission electron micrograph and corresponding elemental maps of Li, C, O, F, and their overlap. (B–D) Electron energy loss spectroscopy spectra of the area in (A) including Li–K edge (B), C–K and O–K edges (C), and F–K and Fe–L_2,3_ edges (D). (E) Low- and high-magnification cryo-scanning transmission electron micrographs and electron energy loss spectroscopy spectra from the marked region, showing the Li–K edge (F), C–K and O–K edges (G), and F–K and Fe–L_2,3_ edges (H).

To better understand the chemical changes underlying the electrochemical performance of CS_Fe_Med, Fe 2p X-ray photoelectron spectroscopy (Figure 8 and Supporting Information, Figure S10) was performed on three representative stages: the as-received powder (CS_Fe_Med_powder, Figure 8A), the pristine electrode (CS_Fe_Med_pristine electrode, Figure 8B), and the post-mortem electrode after extended cycling (CS_Fe_Med_post-mortem electrode, Figure 8C). The fitting was carried out using a physically consistent multiplet model, based on the work by Hughes et al., incorporating defined peak positions, FWHM, and multiplet weightings.^39^ The X-ray photoelectron spectra and the relative abundances of the different Fe species, shown in Figure 8D, reveal a dynamic transformation of the iron oxidation states throughout electrode processing and cycling. The pristine powder is composed primarily of FeO (∼54%, all values represent percentage shares of bonds) and metallic Fe (∼36%), with minor contributions from FeOOH (∼10%), which aligns with the results obtained from X-ray diffraction. Upon electrode formulation (CS_Fe_Med_pristine), where the active material is combined with PVdF, the Fe^0^ fraction increases slightly to ∼43%, while FeO drops to ∼26% and FeOOH remains at ∼12%. Additionally, Fe_3_O_4_ (∼19%) emerges as a new surface phase, likely due to partial oxidation during slurry casting and drying under ambient conditions, resulting in more electrochemically active species. After long-term cycling (CS_Fe_Med_post-mortem), the X-ray photoelectron spectroscopy pattern was very noisy due to low detected iron species on the surface. The fitting and calculation performed revealed that FeO becomes the most dominant species (∼55%), while the metallic Fe content is substantially reduced to ∼17%. At the same time, FeOOH increases to ∼28%, indicating progressive surface reoxidation under electrochemical conditions. The persistent Fe^0^ and dominant FeO in the post-mortem electrode suggest the formation of nanoscale Fe/FeO heterojunctions, which enhance Li^+^ diffusion and catalyze reversible Li_2_O decomposition (Fe^0^ + Li_2_O ↔ FeO + 2Li^+^ + 2e^–^).^40^ This backbone effect is common in conversion materials and can lead to progressive capacity activation.^41^ Fe_3_O_4_ becomes undetectable, suggesting either conversion to other phases or transformation during extended redox cycling.

**Figure 8:**
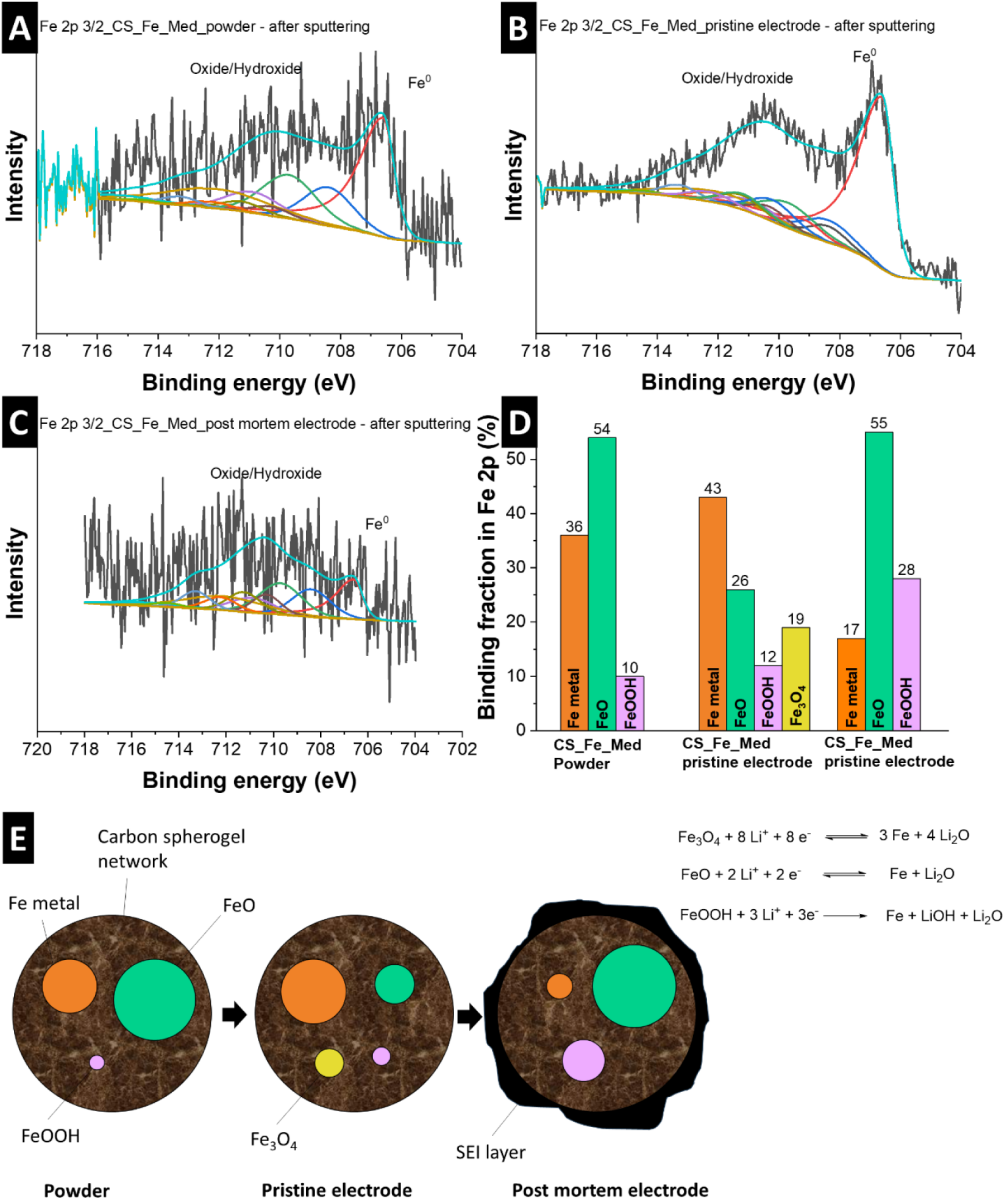
X-ray photoelectron spectra Fe 2p_3/2_ scan of (A) CS_Fe_Med_powder after sputtering, (B) CS_Fe_Med_pristine electrode after sputtering, and (C) CS_Fe_Med_post-mortem after sputtering. (D) graphical demonstration of binding percentage in Fe 2p and (E) schematics of the mechanism of the material in the LIB half-cell.

The systematic increase in oxidized species (Fe^2+^/Fe^3+^) correlates with the observed rise in capacity during extended cycling, indicating enhanced electrochemical activity through a conversion-type mechanism. The persistence of Fe^0^ and FeO, even after 230 cycles, reveals partial reversibility and sustained redox cycling, enabled by the conductive carbon spherogel matrix. The rise in FeOOH may introduce surface-mediated redox reactions (Fe^3+^ + e^–^ ↔ Fe^2+^) at higher potentials, adding nonfaradaic capacity. While FeOOH is typically unstable, its *in situ* generation on conductive carbon or Fe^0^ surfaces could enable sustained activity.^42^ The high oxide-to-metal ratio (4.9) implies extensive electrolyte decomposition, but the LiF/Li_2_CO_3_-rich SEI (common with Fe-based anodes) may improve Li^+^ transport. Concurrently, the catalytic reduction of LiOH (from FeOOH) by Fe^0^ could liberate additional Li^+^, thereby contributing to an increased capacity. These findings align with XRD/Raman data, which reveal progressive amorphization and metallic phase formation, and are corroborated by cryo-STEM-EELS (Figure 7), which identifies LiF, Li_2_CO_3_, and Fe within the SEI (consistent with the ∼262 nm average thickness observed by SEM) and the electrode core. Together, they demonstrate a dynamic reorganization of iron species during cycling, facilitating reversible redox processes and capacity enhancement.

The X-ray photoelectron spectroscopy analysis reveals a critical redox evolution mechanism in the CS_Fe_Med electrode: the *in situ* stabilization of mixed Fe^0^/Fe^2+^/Fe^3+^ states within the carbon framework promotes reaction reversibility, fast kinetics, and highlong-term capacity retention, thereby providing fundamental insights into designing robust conversion-type electrodes through tailored host-guest interactions.

To benchmark the electrochemical performance of the Fe-loaded carbon spherogel electrodes (R-Sphero, Figure 9) against state-of-the-art materials, we compared them to a range of literature-reported iron-based anodes for lithium-ion batteries (Figure 9 and Table 2). The rate capability of CS_Fe_Low (R-Sphero) was assessed alongside different reference materials (RA-RH; Figure 9A). Although the initial specific capacity of RI at 0.1 A g^–1^ is moderately lower than that of most benchmark systems, it displays substantially improved capacity retention with an increasing specific current. Even at high rates (4–8 A g^–1^), the capacity of our CS_Fe_Low electrode decreases only gradually, remaining comparable to, or even surpassing, several reported iron-based electrodes. In contrast, most reference materials exhibit pronounced capacity fading at elevated rates, indicative of kinetic limitations or structural instability. This performance is achieved despite the low content (<20 mass %) of active iron species in the CS_Fe_Low composite, compared to the 80 mass % content in conventional systems. These findings underscore the efficient ion and electron transport pathways within the carbon spherogel matrix, as well as the outstanding structural integrity of the electrode during rapid charge/discharge cycles. The initial capacity of CS_Fe_Low reaches approximately 612 mAh g^–1^ (Figure 9B) without the use of conductive additives (composition: 90 mass % active material, 10 mass % PVdF binder), reflecting the intrinsic conductivity and electrochemical activity of the composite. Notably, while many state-of-the-art materials exhibit higher initial capacities, for instance, 1135 mAh g^–1^ in Fe_2_O_3_ nanosheets reported by Chen et al.^43^ or 1294 mAh g^–1^ in MXene-hollow carbon nanofibers with Fe_3_C in the work by Lu et al.,^44^ these typically suffer from substantial capacity losses, often exceeding 20–30% after limited cycling.

**Figure 9:**
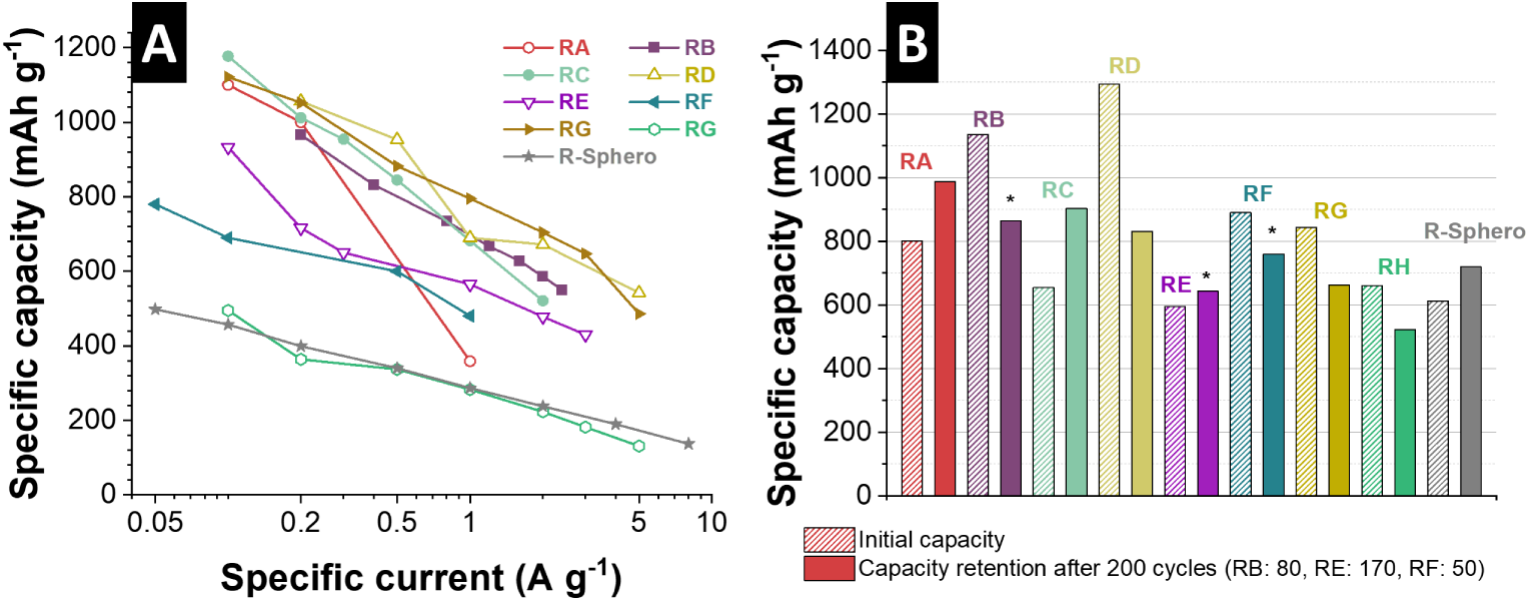
Graphical illustration and overview of the obtained specific capacities after cycling for different iron-based electrodes of the state-of-the-art systems. (A) Comparison of the rate handling performance of different state-of-the-art systems with this work. (B) Comparison of performance stability, comparing the initial capacity and 200^th^ cycle capacity of different state-of-the-art systems with this work. RA: ref. 45; RB: ref. 43; RC: ref. 46; RD: ref. 44; RE: ref. 47; RF: ref. 48; RG: ref. 49; RH: ref. 50; R-Sphero (this work).

**Table 2:** Overview of Electrochemical Performance and Parameters of Different Iron-Based Electrode Materials as Anodes in LIBs^a^

ref.	Identifier Figure 9	Type of active material	Electrode composition	Potential	Electrolyte (all by volume)	Capacity/mAh g^–1^ at rate	Cycles
Wang et al.^45^	RA	Fe_2_O_3_ nanotubes	70% Fe_2_O_3_, 20% AB, 10% PVdF	0.01–3.00 V vs. Li^+^/Li	1 M LiPF_6_ in EC/DMC	988 mAhg^–1^ at 0.2 A g^–1^	250
Chen et al.^43^	RB	Fe_2_O_3_ nanosheets	40% Fe_2_O_3_, 40% AB, 20% PVdF	0.10–3.00 V vs. Li^+^/Li	1 M LiPF_6_ in EC/DMC	865 mAhg^–1^ at 0.2C	80
Chen et al.^46^	RC	Core–shell C@Fe_3_C/Fe	80% C@Fe_3_C/Fe, 10% AB, 10% PVdF	0.01–3.00 V vs. Li^+^/Li	1 M LiPF_6_ in EC/DMC/EMC/DEC (30:15:20:35)	808 mAhg^–1^ at 1 A g^–1^	710
Lu et al.^44^	RD	MXene hollow carbon nanofibers confined with Fe_3_C	100% MXene hollow carbon nanofibers confined with Fe_3_C	0.01–3.00 V vs. Li^+^/Li	1 M LiPF_6_ in DOL/DME	861 mAhg^–1^ at 0.2 A g^–1^	200
Ryu et al.^47^	RE	Fe_3_O_4_/carbon	80% Fe_3_O_4_/carbon, 10% Super P, 5% CMC, 5% SBR	0.01–3.00 V vs. Li^+^/Li	1.3 M LiPF_6_ in EC/EMC/DEC (3/5/2)	644 mAhg^–1^ at 1 A g^–1^	200
Oubla et al.^48^	RF	Fe_3_O_4_@rGO	80% Fe_3_O_4_ @rGO, 10% SuperP, 10% PVdF	0.01–3.00 V vs. Li^+^/Li	1 M LiPF_6_ in EC/DMC	890 mAhg^–1^ at 0.05 A g^–1^	50
Zhao et al.^49^	RG	FeC_2_O_4_	60% FeC_2_O_4_, 30% Super P, 10% PVdF	0.01–3.00 V vs. Li^+^/Li	1 M LiPF_6_ in EC/DEC	900 mAhg^–1^ at 5 A g^–1^	1200
Zhang et al.^50^	RH	FeC_2_O_4_·2H_2_O	60% FeC_2_O_4_, 30% Super P, 10% PVdF	0.01–3.00 V vs. Li^+^/Li	1 M LiPF_6_ in EC/DEC	523 mAhg^–1^ at 0.5 A g^–1^	200
**This work**	**R-Sphero**	**CS_Fe_Low**	**90% CS_Fe_Low, 10% PVdF**	**0.01–3.00 V** **vs. Li** ^**+**^ **/Li**	**1 M LiPF**_**6**_ **in EC/DMC (1:1 by volume)**	**1190** **mAh g** ^**–1**^ **at** **0.1** A g^**–1**^	**300**

a (AB: Acetylene black, PVdF: polyvinylidene fluoride, EC: ethylene carbonate, DMC: dimethyl carbonate, DEC: diethylene carbonate, EMC: ethyl methyl carbonate, DOL: 1,3-dioxolane, DME: dimethoxyethane).

In contrast, our CS_Fe_Low electrode demonstrated robust long-term cycling stability (Figure 9B). Following an initial capacity of 612 mAh g^–1^, a gradual increase to 720 mAh g^–1^ is observed after 200 cycles, corresponding to a capacity enhancement of approximately 18%. Such self-improving behavior during cycling has also been reported in the literature. Comparable trends were only observed in a few studies, such as Fe_2_O_3_ nanotubes in the studies of Wang et al.,^45^ showing an increase from 800 mAh g^–1^ to 987 mAh g^–1^ over 200 cycles, and the core–shell C@Fe_3_C/C material described by Chen et al.,^46^ with capacities rising from 655 mAh g^–1^ to 903 mAh g^–1^. Further, after 300 cycles, our CS_Fe_Low achieves a capacity of 1190 mAh g^–1^, surpassing the performance of all referenced materials at their respective reported cycle numbers. CS_Fe_Low thus distinguishes itself by offering a balanced performance profile. While its absolute capacity is lower at initial stages, the superior rate retention and progressive capacity increase under prolonged cycling reflect highly effective charge transport and structural robustness within the carbon spherogel framework. In summary, although our CS_Fe_Low material does not deliver the highest initial specific capacity, it provides a sustainable, binder-only, and additive-free design combined with high rate capability and long-term cycling stability, emphasizing its potential for practical application in lithium-ion energy storage systems.

## 4 Conclusions

We report the synthesis of iron-loaded hybrid carbon spherogels via a polystyrene-templated resorcinol-formaldehyde (RF) sol-gel route, adapting our previously established method for titania hybrids. Iron lactate precursors were incorporated at varying concentrations (10–30 mass %) to yield monolithic spherogels (CS_Fe_Low/Med/High) after carbonization at 800 °C under argon. The resulting materials feature uniform, interconnected hollow carbon spheres (170 ± 4 nm diameter, 10 ± 1 nm wall thickness) with embedded iron nanoparticles (10–30 nm), as confirmed by scanning electron micrographs/scanning transmission electron microscopy, and EDX. Structural characterization revealed graphitic carbon frameworks and metallic Fe^0^ phases, with higher iron loadings promoting crystallinity. Electrochemically, all samples exhibited reversible Fe^0^/Fe^3+^ redox activity and an increasing Li-ion storage capacity. XPS analysis elucidated the dynamic evolution of iron speciation during cycling. While the pristine electrode contained a mix of Fe^0^ (43%), FeO (26%), FeOOH (12%), and Fe_3_O_4_ (19%), postcycling measurements revealed a compositional shift toward FeO (55%) and FeOOH (28%), alongside residual Fe^0^ (17%). This oxidation progression, evidenced by the rising oxide-to-metal ratio (from 1.3 to 4.9), aligns with the observed capacity increase and suggests a dual mechanism: (i) partial reversibility of Fe^0^/FeO interfaces and (ii) pseudocapacitive contributions from FeOOH surface redox. The sample with the highest Fe delivered the highest initial capacity (619 mAh g^–1^), while the sample with the lowest Fe loading variant showed superior cycling stability (194% capacity retention after 300 cycles). After 300 cycles, our best-performing electrode material achieved a specific capacity of 1190 mAh g^–1^, outperforming most state-of-the-art iron materials. Post-mortem analyses identified a LiF-dominated SEI layer and preservation of Fe^0^ nanoparticles, elucidating the interplay among iron content, structural integrity, and performance. This work highlights the tunability of iron-carbon spherogels for LIB anodes, achieving a balance between high capacity and long-term stability through controlled precursor loading.

## Supplementary Material



## Data Availability

The data can be made available upon request.

## Notes

Scanning electron micrographs and transmission electron micrographs of CS_Fe_Low, CS_Fe_Med, and CS_Fe_High; elemental mapping and EDX spectra; dynamic light scattering of polystyrene spheres; nitrogen gas sorption isotherms of CS_Fe_Low, CS_Fe_Med, and CS_Fe_High; additional post-mortem analysis via X-ray diffraction, scanning electron micrographs, X-ray photoelectron spectroscopy measurements, and transmission electron micrographs of CS_Fe_Med pristine, after 1 cycle, after 23 cycles, and after 230 cycles (PDF)
