# Supplementary material for: Iron-Loaded Carbon Spherogels as Sustainable Electrode Materials for High-Performance Lithium-Ion Batteries
Source: Chem Mater. 2026 Jan 29;38(5):2237–52. doi: 10.1021/acs.chemmater.5c02442 (PMC12980705; doi:10.1021/acs.chemmater.5c02442)
Supplement: Supplementary file 1 [file cm5c02442_si_001.pdf]

## Supporting Information

# Iron-Loaded Carbon Spherogels as Sustainable Electrode Materials for High-Performance Lithium-Ion Batteries

*Saeed Borhani,[1] Le Thi Thao,[2,3] Gregor A. Zickler,[1] Antje Quade,[4]*
*Michael S. Elsaesser,[1,*] Volker Presser,[2,3,5,*] Stefanie Arnold,[3,*]*

[1] *Chemistry and Physics of Materials, University of Salzburg, 5020 Salzburg, Austria*

[2] *INM - Leibniz Institute for New Materials, Campus D2 2, 66123, Saarbrücken, Germany*

[3] *Department of Materials Science & Engineering, Saarland University, Campus D2 2, 66123, Saarbrücken, Germany*

[4] *Leibniz Institute for Plasma Science and Technology, Felix-Hausdorff-Straße 2, 17489 Greifswald, Germany*

[5] *saarene - Saarland Center for Energy Materials and Sustainability, Campus C4 2, 66123 Saarbrücken, Germany*

* Corresponding authors:

- michael.elsaesser@plus.ac.at (MSE)
- volker.presser@leibniz-inm.de (VP)
- stefanie.arnold@uni-saarland.de (STA)

## Supporting Tables

**Table S1:** Energy-dispersive X-ray elemental analysis of pristine CS_Fe_Med electrode, after 1 completed cycle, after 23 and 230 cycles. All values are given in mass%. Values below the detection limit are noted as "/"

| | **C** | **F** | **Fe** | **O** | **P** | **Si** | **Cl** |
|---|---|---|---|---|---|---|---|
| Pristine CS_Fe_Med | 71.5 | 5.0 | 4.5 | 4.4 | / | 0.1 | 0.1 |
| After 1 cycle | 24.4 | / | 1.2 | 22.4 | 0.3 | / | 0.3 |
| After 23 cycles | 34.1 | 9.9 | 1.7 | 30.2 | 1.9 | / | / |
| After 230 cycles | 37.1 | 7.3 | 1 | 46.6 | 1.1 | 0.2 | / |

## Supporting Figures

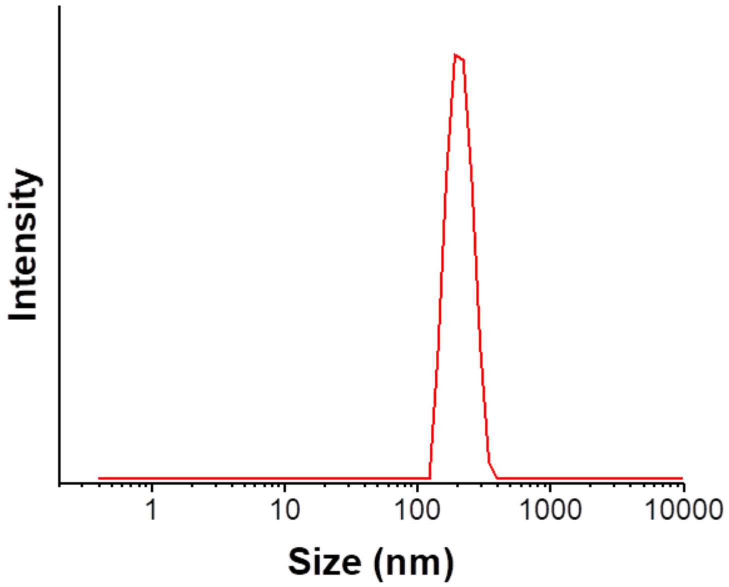


**Figure S1**: Dynamic light scattering diagram of polystyrene spheres used as templates for the synthesis of iron-loaded carbon spherogels.

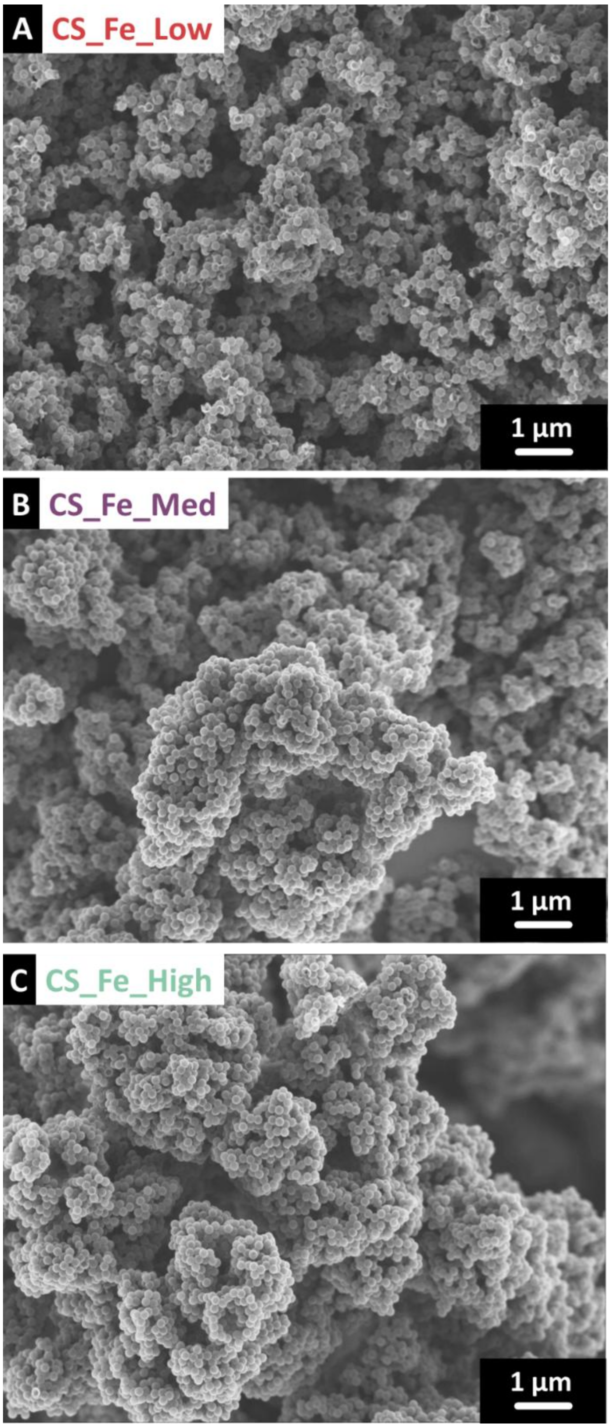


**Figure S2:** Scanning electron micrograph of (**A**) CS_Fe_Low, (**B**) CS_Fe_Med, (**C**) CS_Fe_High.

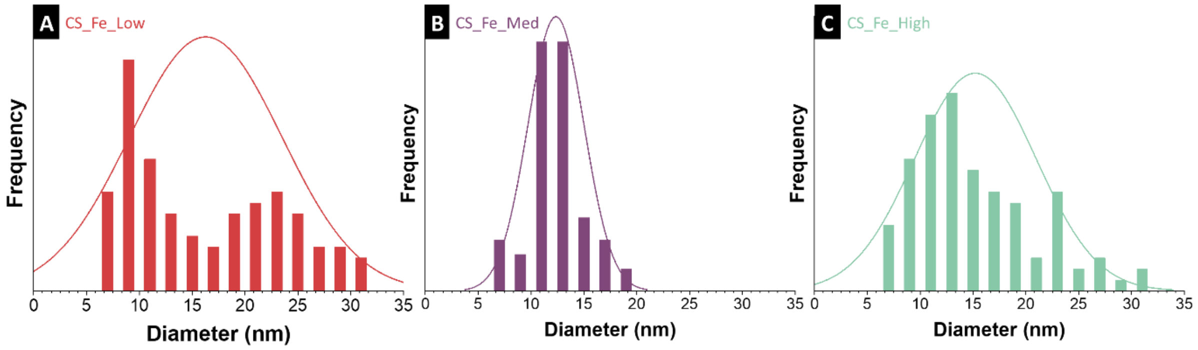


**Figure S3**: Size distribution histograms of iron-based nanoparticles in (**A**) CS_Fe_Low, (**B**) CS_Fe_Med, and (**C**) CS_Fe_High, obtained by measuring 100 nanoparticles per sample using ImageJ software.

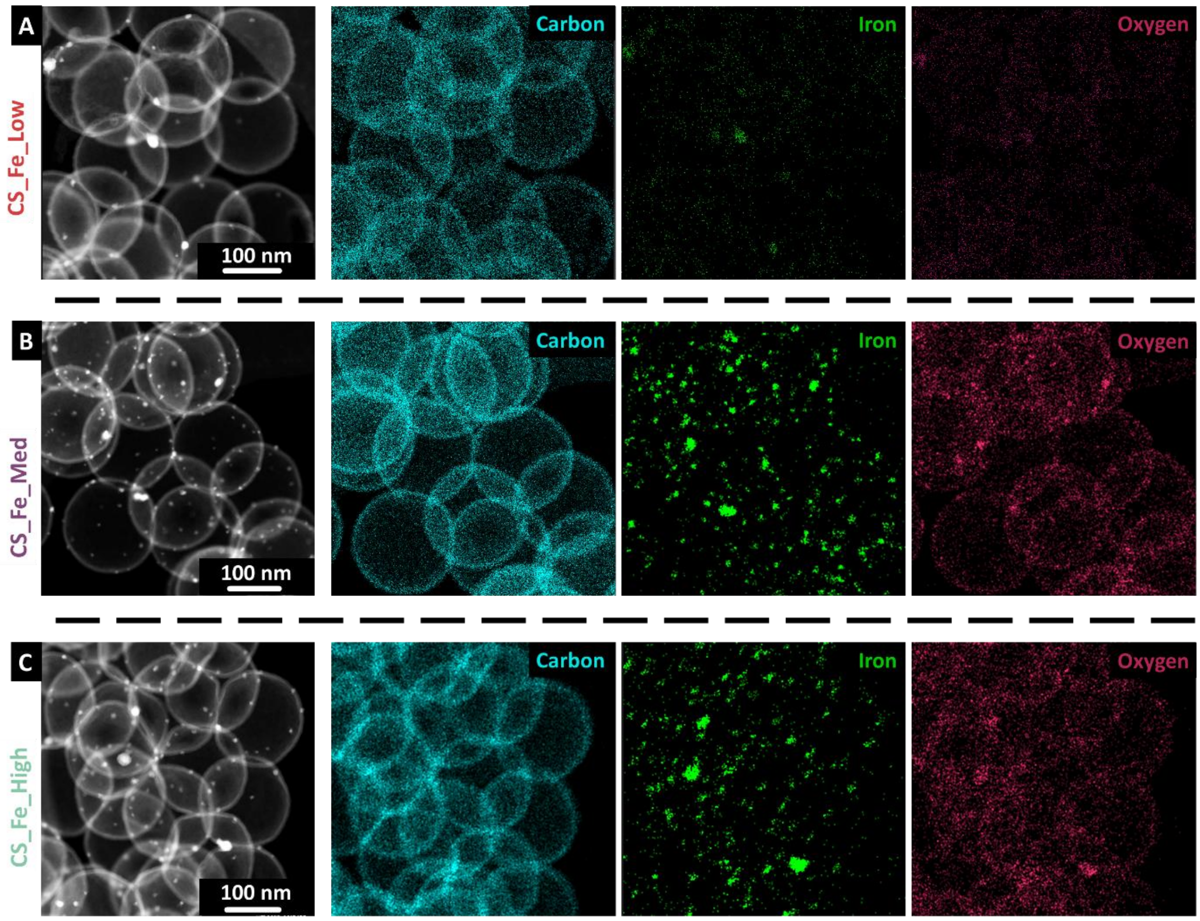


**Figure S4:** Scanning transmission electron micrographs and energy-dispersive X-ray spectroscopy mapping (**A**) CS_Fe_Low, **(B**) CS_Fe_Med, (**C**) CS_Fe_High.

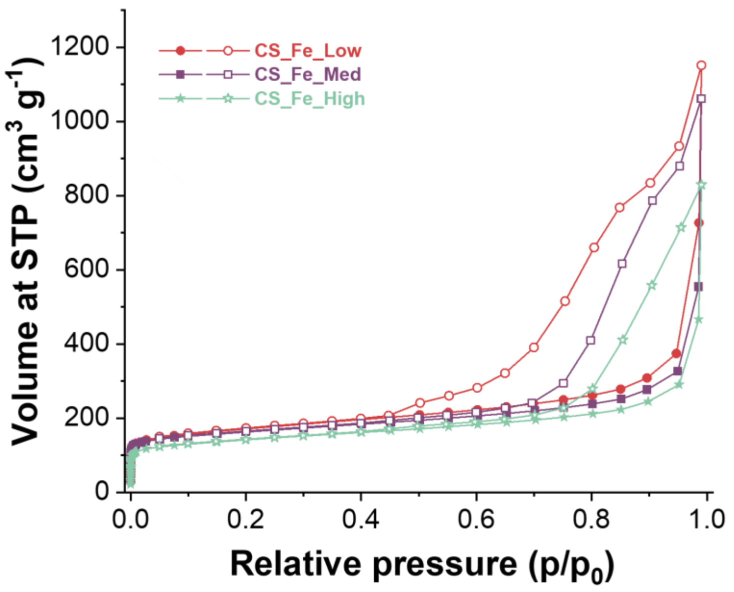


**Figure S5**: Nitrogen sorption isotherms at -196 °C of iron-loaded carbon spherogels with different iron contents (CS_Fe_Low, CS_Fe_Med, CS_Fe_High). STP: Standard temperature and pressure.

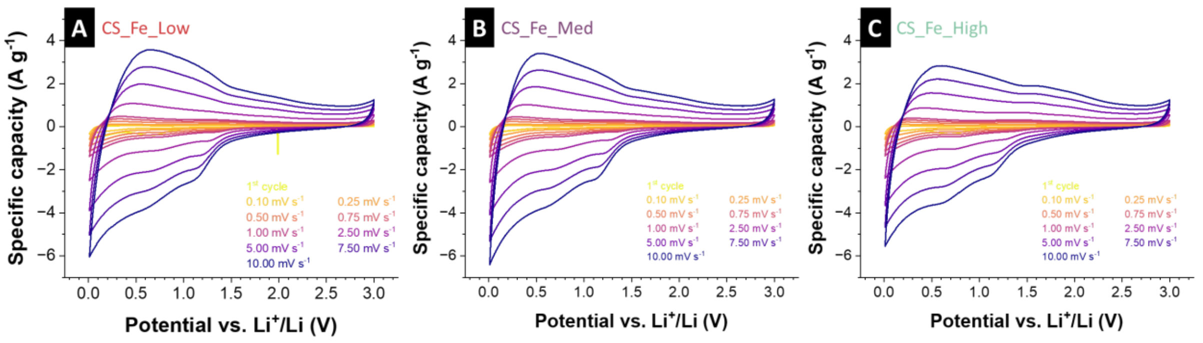


**Figure S6**: Electrochemical characterization of hybrid iron-loaded carbon spherogels showing cyclic voltammograms at different scan rates for each 5 cycles for (**A**) CS_Fe_Low, (**B**) CS_Fe_Med, and (**C**) CS_Fe_High.

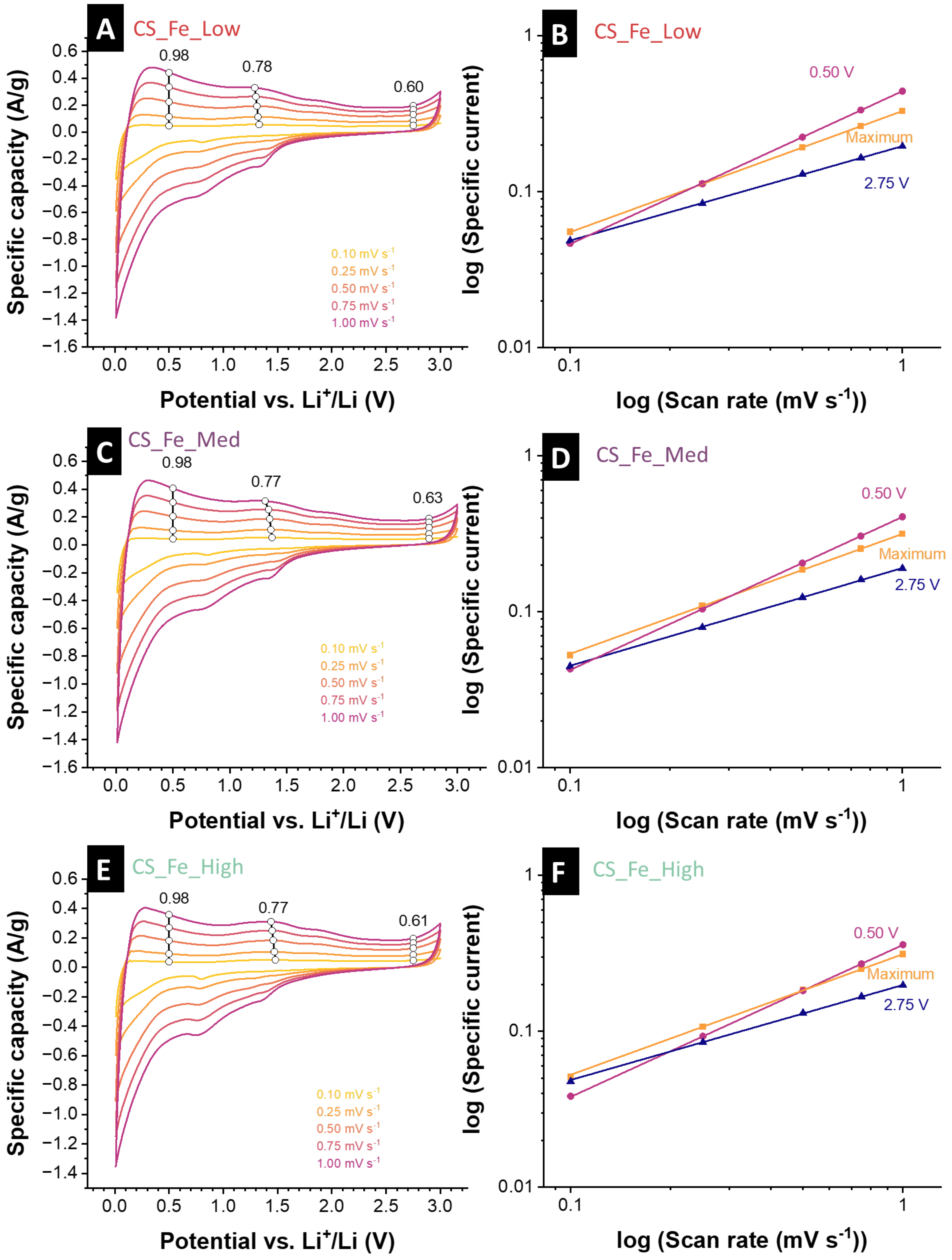


**Figure S7**: Cyclic voltammograms at different rates and kinetic fitting to calculate *b*-values for (**A**-**B**) CS_Fe_Low, (**C**-**D**) CS_Fe_Med, and (**E**-**F**) CS_Fe_High.

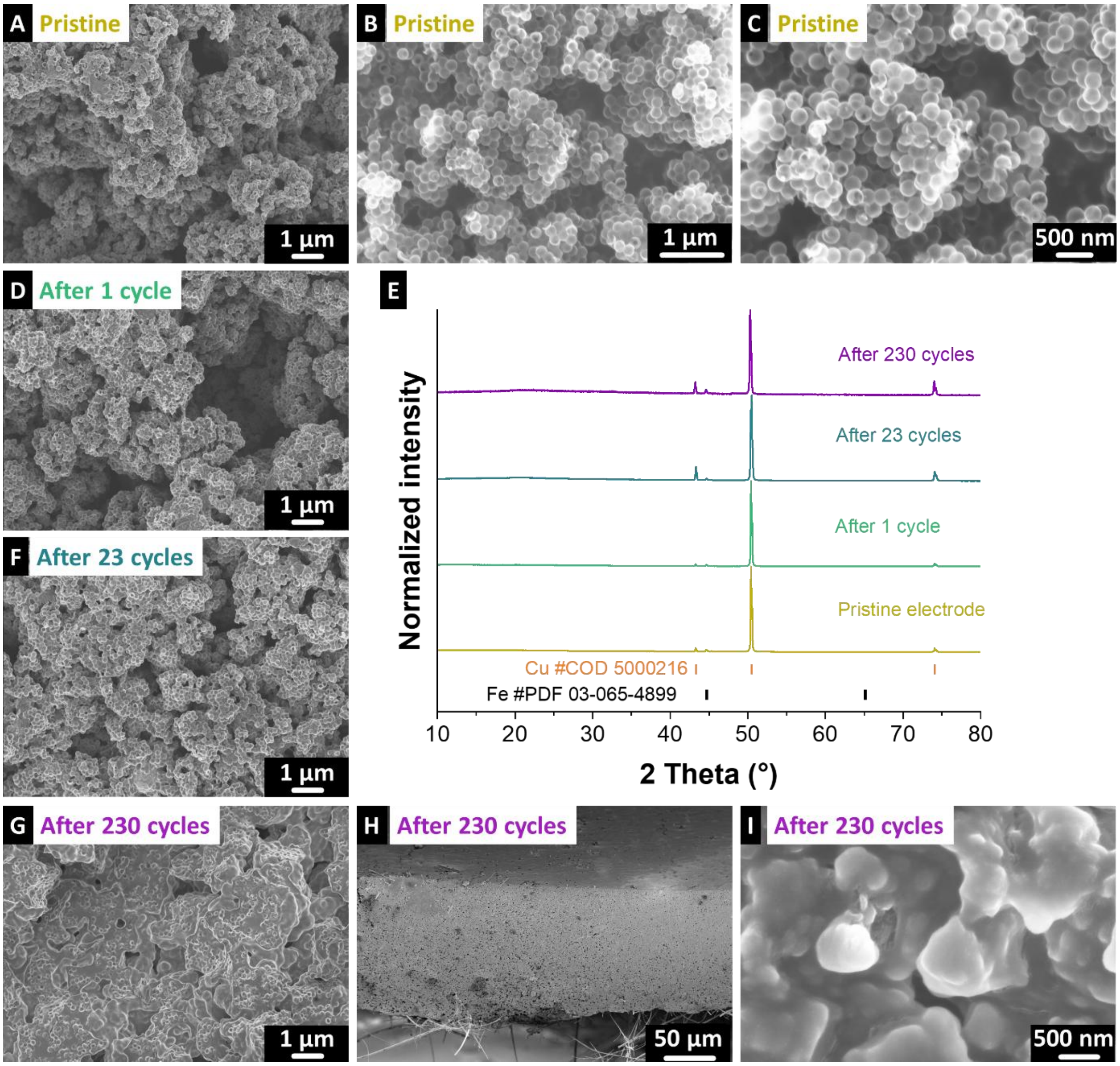


**Figure S8**: Post-mortem analysis showing scanning electron micrograph of (**A-C**) CS_Fe_Med pristine electrode, (**D**) CS_Fe_Med after 1 completed cycle, (**F**) CS_Fe_Med after 23 cycles, and (**G**) CS_Fe_Med after 230 cycles. (**E**) X-ray diffractograms of CS_Fe_Med pristine electrode, CS_Fe_Med after 1 completed cycle, CS_Fe_Med after 23 cycles, and CS_Fe_Med after 230 cycles with the copper current collector.

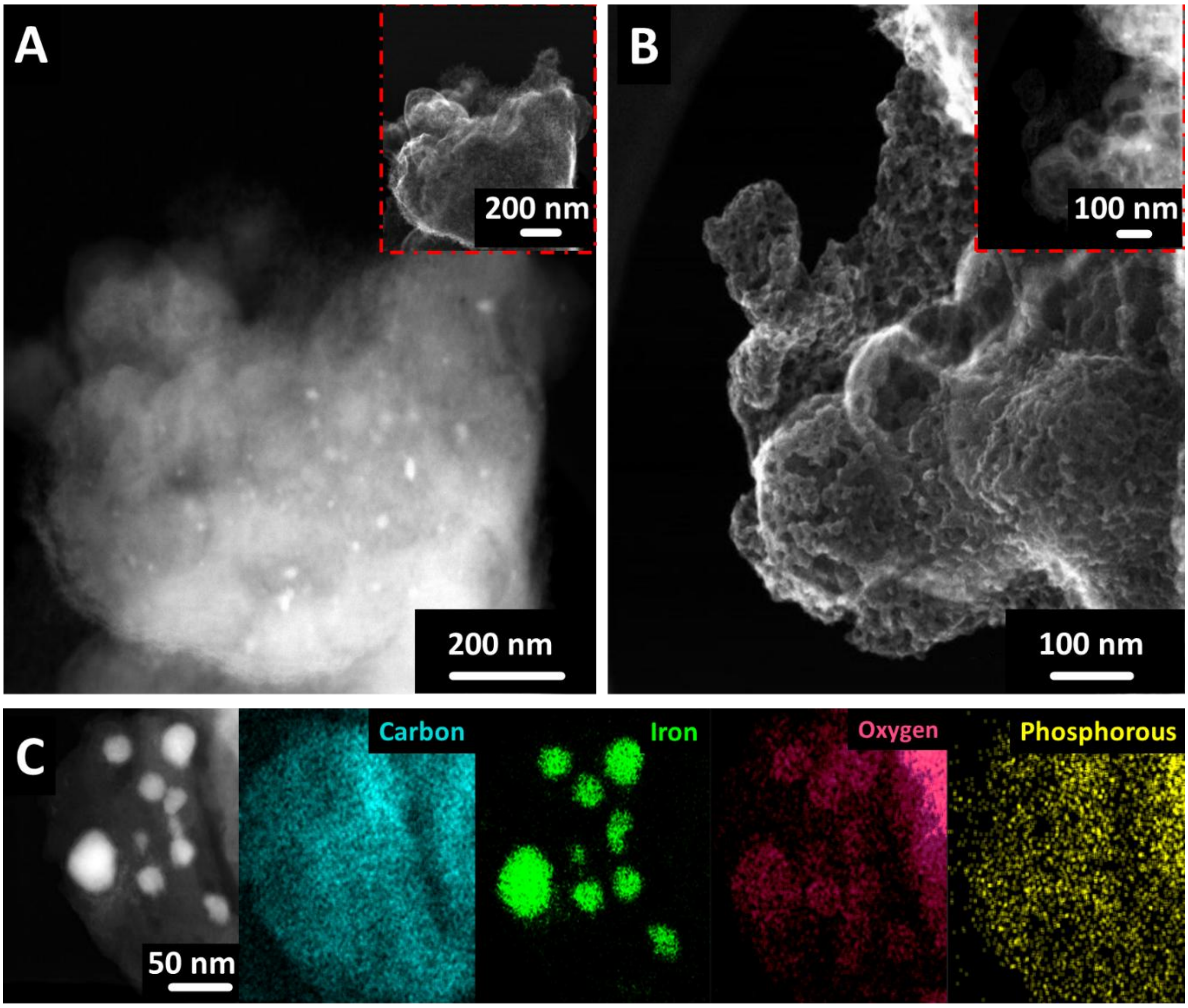


**Figure S9**: Scanning transmission electron micrographs and energy-dispersive X-ray spectroscopy mapping of CS_Fe_Med after 230 cycles.

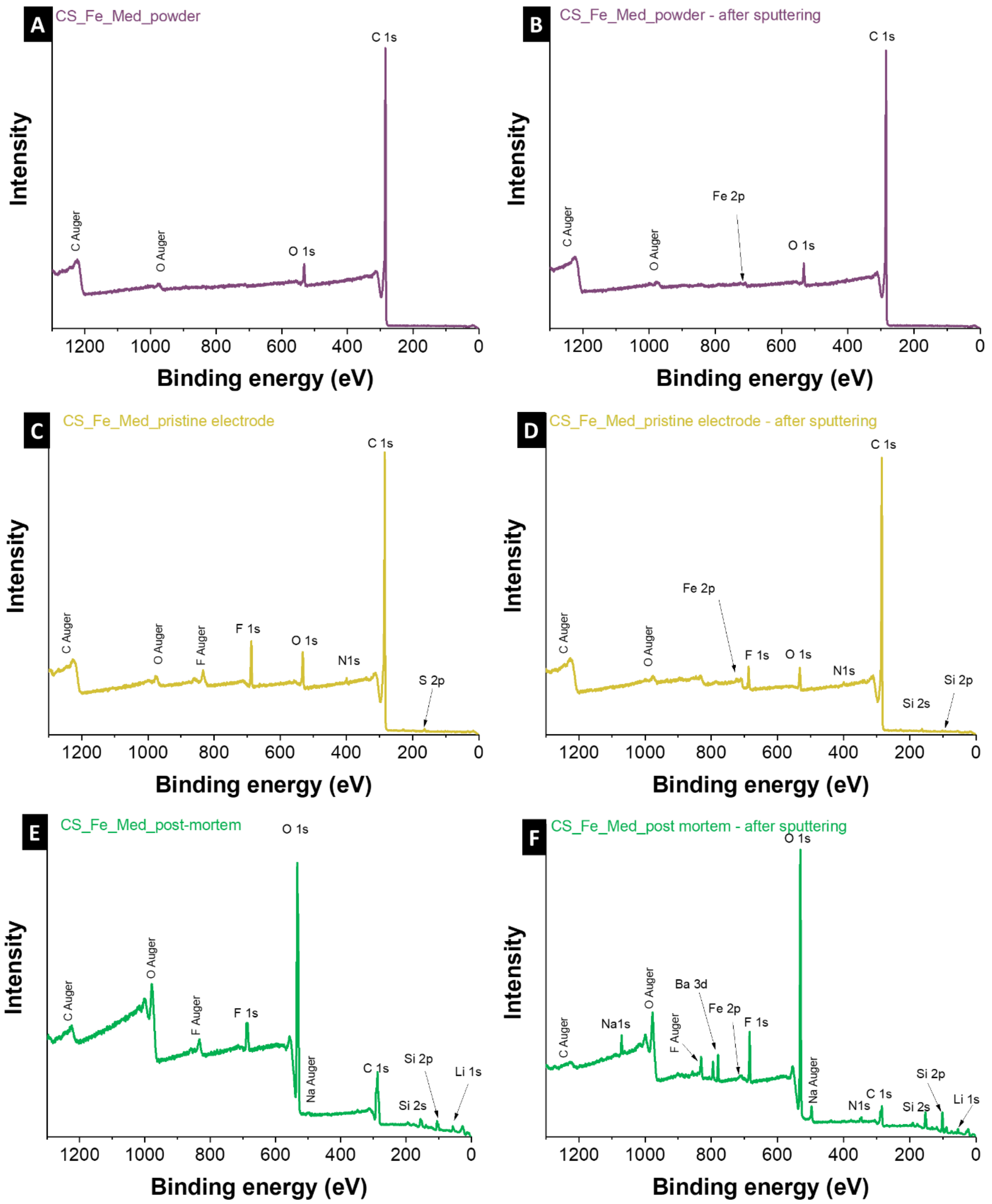


**Figure S10**: X-ray photoelectron spectra survey scans using Al-Kα radiation for (**A**) CS_Fe_Med_powder, (**B**) CS_Fe_Med_powder after sputtering, (**C**) CS_Fe_Med_pristine electrode, (**D**) CS_Fe_Med_pristine electrode after sputtering, (**E**) CS_Fe_Med_post-mortem, and (**F**) CS_Fe_Med_post-mortem after sputtering.
